# Impacts of incorporating personal genome sequencing into graduate genomics education: a longitudinal study over three course years

**DOI:** 10.1186/s12920-018-0319-0

**Published:** 2018-01-30

**Authors:** Michael D. Linderman, Saskia C. Sanderson, Ali Bashir, George A. Diaz, Andrew Kasarskis, Randi Zinberg, Milind Mahajan, Sabrina A. Suckiel, Micol Zweig, Eric E. Schadt

**Affiliations:** 1 Icahn Institute for Genomics and Multiscale Biology, Icahn School of Medicine at Mount Sinai, New York, NY USA; 2 Department of Genetics and Genomic Sciences, Icahn School of Medicine at Mount Sinai, New York, NY USA; 3 Health Behaviour Research Centre, Department of Epidemiology and Public Health, University College London, London, UK; 4 Department of Clinical Genetics, Great Ormond Street Hospital, London, UK; 5 Department of Computer Science, Middlebury College, Middlebury, VT USA

**Keywords:** Genomics education, Personal genome sequencing, Whole genome sequencing

## Abstract

**Background:**

To address the need for more effective genomics training, beginning in 2012 the Icahn School of Medicine at Mount Sinai has offered a unique laboratory-style graduate genomics course, “Practical Analysis of Your Personal Genome” (PAPG), in which students optionally sequence and analyze their own whole genome. We hypothesized that incorporating personal genome sequencing (PGS) into the course pedagogy could improve educational outcomes by increasing student motivation and engagement. Here we extend our initial study of the pilot PAPG cohort with a report on student attitudes towards genome sequencing, decision-making, psychological wellbeing, genomics knowledge and pedagogical engagement across three course years.

**Methods:**

Students enrolled in the 2013, 2014 and 2015 course years completed questionnaires before (T1) and after (T2) a prerequisite workshop (*n* = 110) and before (T3) and after (T4) PAPG (*n* = 66).

**Results:**

Students’ interest in PGS was high; 56 of 59 eligible students chose to sequence their own genome. Decisional conflict significantly decreased after the prerequisite workshop (T2 vs. T1 *p* < 0.001). Most, but not all students, reported low levels of decision regret and test-related distress post-course (T4). Each year baseline decisional conflict decreased (*p* < 0.001) suggesting, that as the course became more established, students increasingly made their decision prior to enrolling in the prerequisite workshop. Students perceived that analyzing their own genome enhanced the genomics pedagogy, with students self-reporting being more persistent and engaged as a result of analyzing their own genome. More than 90% of respondents reported spending additional time outside of course assignments analyzing their genome.

**Conclusions:**

Incorporating personal genome sequencing in graduate medical education may improve student motivation and engagement. However, more data will be needed to quantitatively evaluate whether incorporating PGS is more effective than other educational approaches.

**Electronic supplementary material:**

The online version of this article (doi: 10.1186/s12920-018-0319-0) contains supplementary material, which is available to authorized users.

## Background

Seeking to address the growing gap between the demand for genome sequencing and the supply of genomics professionals with the necessary training [1–10], in 2012 a multidisciplinary group of faculty at the Icahn School of Medicine at Mount Sinai developed, and have offered yearly since, a novel laboratory-style medical genomics course, “Practical Analysis of Your Personal Genome” (PAPG). The objective is to prepare future clinicians and researchers to effectively employ next generation sequencing (NGS) and, in particular, whole genome sequencing (WGS), around which we expect genomic medicine will ultimately converge. PAPG students uniquely have the opportunity, if they so choose, to sequence and analyze their own whole genome at no cost to them [11]. The motivating hypothesis was that for those students who desire to do so, the opportunity to analyze their own genome would improve educational outcomes by increasing their engagement with the course pedagogy and their motivation to master the complexity of genome analysis.

There are potential risks to accessing personal genome sequencing (PGS) results, particularly in an educational setting where distress could adversely impact the student’s wellbeing and their learning [12–15]. The PAPG course sequence and sequencing protocol [11] were designed to promote informed decision-making, minimize the potential for test-related distress and maximize the educational value of incorporating PGS. To assess the protocol for and impacts of incorporating PGS into graduate genomics education, a companion research study has evaluated students’ attitudes towards, decision-making for, and the outcomes of incorporating PGS into PAPG.

This manuscript expands upon previously reported results from the initial PAPG student cohort (*n* = 19) [16, 17]. In 2012, students’ baseline interest in sequencing their own genome was high, but many students expressed decisional conflict. Decisional conflict decreased after a prerequisite workshop, “Introduction to Human Genome Sequencing” (IHGS), that is part of the PAPG informed decision-making protocol, indicating that students perceived their decision as more informed after completing this pre-decision preparatory coursework [16]. Post-PAPG, most, but not all, of the 2012 students reported low levels of decision regret and test-related distress [17]. In follow-up interviews, students reported that the opportunity to analyze their own genome positively contributed to their motivation and engagement [17].

The PAPG course has been offered yearly since 2012 and is now an established component of multiple training programs. Here we report on the attitudes towards genome sequencing, decision-making for PGS and psychological and educational outcomes of the 110 students enrolled in the 2013, 2014 and 2015 cohorts, comprising 59 students eligible to sequence their own genome, and a new comparison group of 51 primarily enrolled only in the prerequisite workshop. In these subsequent years we employed expanded questionnaires that incorporated a more relevant knowledge measure and new quantitative measures of engagement. This much larger cohort improves our understanding of the impacts of incorporating PGS into genomics pedagogy and represents one of the larger study cohorts to undergo predispositional WGS in any setting, not just education [18].

Our second aim was to evaluate changes between course years as the course, and WGS more generally, have become more established. Students in 2013-onwards had one or more years to anticipate enrolling in the course and contemplate the decision to sequence their own genome or not. And with the increasing use of genome sequencing in both research and clinical settings, students are increasingly likely to have encountered this technology prior to enrolling in the PAPG course sequence. We hypothesized that students’ attitudes towards genome sequencing and their decision-making for PGS would be different by year as a result. This extended experience will inform how we evaluate courses of this kind and suggest trends that may also be observed among participants in predispositional PGS in non-educational settings.

## Methods

### Study and course design

This was a quantitative longitudinal cohort study in which an evolving set of anonymous questionnaires were administered to students enrolled in the “Practical Analysis of Your Personal Genome” (PAPG) course, and its prerequisite workshop “Introduction to Human Genome Sequencing” (IHGS), at the Icahn School of Medicine at Mount Sinai in the years 2013, 2014 and 2015. The study protocol, course design and approval process are described in more detail in related publications [11, 16, 17]. In brief, questionnaires were administered at 4 time points: before (T1) and immediately after (T2) IHGS, a two-day 15-h prerequisite workshop, and before (T3) and immediately after (T4) the semester-length PAPG course. The course sequence and study time line is summarized in Additional file 1: Figure S1. Paper questionnaires were administered in 2013 and transitioned to the online SurveyMonkey service for 2014 onwards.

As described in related publications, the Institutional Review Board at the Icahn School of Medicine at Mount Sinai determined the sequencing component of PAPG to not be research and this study (#12–1273) to be exempt under category 2 [11, 16, 17]. Electronic consent was obtained from all participants at the beginning of each survey.

PAPG was a required course for students in the Masters of Science in Genetic Counseling program (MSGC) and offered as an elective course to other graduate students, medical students and clinical trainees. A maximum of 20 students per year enrolled in PAPG with the option to obtain and analyze their own whole genome sequence. We describe these students as “genome eligible”. Starting in 2014, students could also enroll in PAPG without the option to obtain their own genome. All PAPG students were required to complete the prerequisite IHGS workshop. Additional seats in IHGS were opened to all students, faculty and employees of Mount Sinai Medical Center up to a maximum total enrollment of approximately 35 students per year. Thus approximately 15 students per year were not continuing onto PAPG and or not eligible to obtain their own genome sequence (these students knew that they would not be able to obtain their own genome sequence when they enrolled); these participants, described as “genome ineligible”, serve as a comparison group for pre-test measures of interest, attitudes and psychological wellbeing. Enrollment and response rates are listed in Table 1; enrollment demographics are described in Additional file 1: Table S3.

**Table 1:** Enrollment and survey response rate by year and time point. The numbers of PAPG students enrolled without the option to sequence their own genome are shown in parentheses

	2013		2014		2015	
	IHGS Only	IHGS + PAPG	IHGS Only	IHGS + PAPG	IHGS Only	IHGS + PAPG
Enrolled	19	19^a^	10	25 (5)^b^	15	22 (2)^c^
T1	15 (79%)	19 (100%)	10 (100%)	24 (96%)	10 (66%)	21 (95%)
T2	12 (63%)	16 (84%)	9 (90%)	23 (92%)	8 (53%)	19 (86%)
T3	N/A	17 (89%)	N/A	24 (96%)	N/A	18 (82%)
T4	N/A	15 (79%)	N/A	21 (84%)	N/A	17 (77%)

^a^Two of these students dropped the course during the semester

^b^Two of the students who enrolled without the option to obtain their genome dropped the course during the semester

^c^One of the students who enrolled without the option to obtain their genome dropped the course during the semester

As shown in the course timeline in Additional file 1: Figure S1 students make the decision to undergo whole genome sequencing after T2 (before T3) and obtain their genome sequence data (or a reference genome) after T3. Thus the T1 and T2 time points are “pre-decision”, T3 is “post-decision” but “pre-results” and T4 is “post-results”. This timeline differs from the previously reported 2012 cohort who made their decision after T3 [16]. PAPG students do not receive a report with a specific set of genetic findings; instead they receive the raw sequencing data and analyze it themselves in a structured way during the course. Thus the only results they “receive” are those they themselves generate. A student can choose to obtain their genome data but not actually view the data. As part of the course pedagogy PAPG students can create optional exclusion regions that mask variant calls in those genomic regions (described in more detail in [11]). The number of students reporting using exclusion regions, the time they reported spending analyzing their genomic data and the kinds and number of genomic findings they reported generating are described in the Results section.

### Measures

The questionnaires assessed students’ motivations for choosing to (not) sequence their own genome, decision-making, attitudes towards personal genome sequencing inside and outside the classroom, psychological wellbeing, actions, and self-rated and objectively measured genomics knowledge. Decision-related variables were assessed with the Decisional Conflict Scale (DCS) [19], the Decision Regret Scale (DRS) [20] and the Satisfaction with Decision Scale (SWD) [21]. Interests and attitudes were assessed with measures adapted from Ormond et al. [22] and measures developed for this study [16, 17]. Depression was assessed with the 10-question version of the Center for Epidemiologic Studies Depression Scale (CES-D 10) [23], anxiety with the short-form State-Trait Anxiety Inventory (STAI-6) [24] and test-related distress with an adapted version of the Multidimensional Impact of Cancer Risk Assessment (MICRA) [25]. Actions, such as use of exclusions, were assessed with questions developed specifically for this study. Self-rated knowledge was assessed with a measure adapted from the HealthSeq WGS study [26] and objective knowledge with a novel multiple choice test developed for this study. A complete listing of all measures, including any modifications to the published measure or scoring method, is included in the Supplemental Methods and Data [Additional file 1] along with copies of the survey instruments [Additional files 2, 3 and 4].

### Data analysis

All data analysis was performed using the R statistical package version 3.2. Missing data was omitted. The Wilcoxon signed-rank test was used to compare differences between pairs of time points for ordinal data. Kendall’s tau was used to evaluate bivariate correlations. Repeated measures ANOVAs were performed with linear models. The Kruskal-Wallis or Wilcoxon-Mann-Whitney test (ordinal, for 3 and 2 years respectively) and Chi-square test (categorical) were used to evaluate differences between course years within a single time point. To account for multiple hypothesis testing, *p*-values ≤0.001 were considered significant [27]. Effect sizes were computed using *r* for ordinal data and ϕ for categorical data, and were described using Cohen’s criteria of 0.1 for small effect, 0.3 for medium effect and 0.5 for large effect [28].

## Results

### Student characteristics

Table 1 summarizes enrollment and questionnaire response rates for 2013–2015. Students who enrolled in PAPG but were not eligible to sequence their own genome are shown in parentheses. Additional file 1: Table S3 summarizes enrollments by student program and background, e.g. MSGC or post-doctoral fellows.

### Interest, motivations and decision-making before and after prerequisite workshop

Across the three course years 56 of 59 eligible students chose to sequence their own genome. Given the small number of students who chose a reference genome, we do not describe students’ choices by year or demographics to maximize the confidentiality of their choice.

**Fig. 1:**
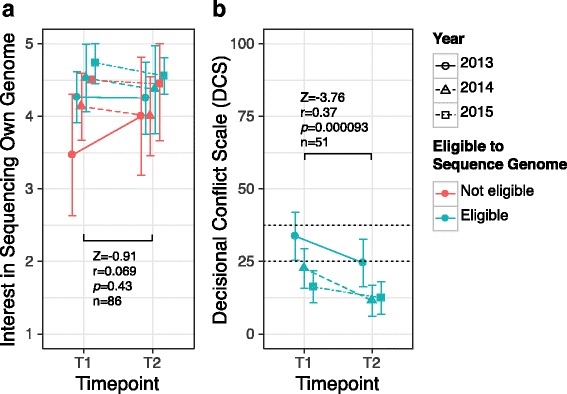
Interest and decisional conflict before and after prerequisite course. Mean and 95% confidence interval for: **a** interest in sequencing own genome, measured on a 1–5 scale from “No, definitely” to “Yes, definitely”, and **b** decisional conflict (DCS) across time point, course year and eligibility to sequence own genome. Vertical axes show scale range. Wilcoxon-signed rank test between time points for all students is shown in each panel. DCS in panel **b** is annotated with cutoffs associated with implementing a decision (< 25) and feeling unsure about a decision (> 37.5) [29]

Figure 1 shows students’ interest in sequencing their own genome and decisional conflict (DCS), before (T1) and after (T2) the prerequisite workshop by year and genome eligibility. Additional file 1: Table S4 summarizes the DCS subscales.

A significant decrease in decisional conflict in genome eligible students was observed after completion of the prerequisite workshop, with the mean score after the prerequisite workshop for all years (and overall) at or less than 25, the threshold associated with implementing a decision [29]. For those students with a baseline DCS score < 25 there is no significant change in DCS score after the prerequisite workshop (Z = −0.42, *p* = 0.68, *r* = 0.06, *n* = 26), while for those students with a baseline DCS score ≥ 25, there was a large effect (Z = −4.11, *p* = 2.3e-6, *r* = 0.58, *n* = 25). Only one of 27 students with a baseline DCS score < 25 had a T2 DCS score ≥ 25. Four students in total had DCS scores ≥37.5 at T2, the threshold associated with feeling unsure about a decision [29].

Figure 1 suggests that there are differences in baseline DCS scores (i.e. at T1) between years and also in the change in DCS scores between T1 and T2. Performing a repeated measures ANOVA, we observed significant negative effects for the course (F(1,48) = 16.6, *p* = 0.00017), and course year (F(2,48) = 9.18, *p* = 0.00042), but not significant effects for the interaction (F(2,48) = 2.08, *p* = 0.14). These results suggest there is no significant difference in the impact of the prerequisite workshop on decisional conflict between course years.

To assess their motivations at T1 and T2, students were asked to indicate their agreement or disagreement with a set of statements about the potential benefits for and concerns about sequencing and analyzing their own genome. The results are summarized in Additional file 1: Table S5 for genome eligible students. At baseline the median student agreed that PAPG would be an opportunity to “get information that would help improve my health” and that their own genome would help them understand genetics concepts better. And the median student strongly agreed that PAPG would be an opportunity to “get a service that I would not ordinarily get if I had to pay full price”. The median student also agreed that they were concerned that they “might get some results what would be disturbing”, but disagreed or were neutral to the other proposed concerns.

While generally there was little significant change in students’ concerns over the prerequisite workshop among genome eligible students, we did observe a significant decrease with medium effect size in agreement with “I feel that I would be at a disadvantage to my classmates if I did not undergo the testing” (Z = −3.35, *p* = 0.00073, *r* = 0.33, *n* = 52). After the introductory course, the median student disagreed with the statements “I would be concerned that my professors would know who took up the offer of testing and who did not” and “I would be concerned that my classmates would know who took up the offer of testing and who did not**”,** with no students agreeing with the former.

### Satisfaction with decision and decision regret

**Fig. 2:**
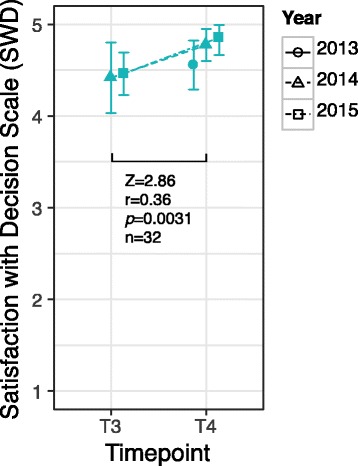
Satisfaction with decision. Mean and 95% confidence interval for satisfaction with decision (SWD) of genome eligible students before and after PAPG by course year. Wilcoxon-signed rank test between time points is shown

Figure 2 shows satisfaction with decision (SWD) before (T3) and after (T4) PAPG by year for genome eligible students. Mean (SD) scores on the Decision Regret Scale (DRS) after PAPG (T4) were 6.46 (10.1) with a range of 0–35. Students were generally satisfied with their decision, with the mean SWD increasing after the PAPG course (mean SWD at T4 was greater than 4.5 in all years; the scale has a range of 1–5 where 5 indicates high satisfaction). We did not observe a significant relationship between DCS at T2 and SWD (τ = −0.26, *p* = 0.037, *n* = 42) or DRS (τ = 0.29, *p* = 0.015, *n* = 43).

### Impact of personal genome sequencing in PAPG

#### Analyses performed and genetic results obtained

Table 2 summarizes whether students who sequenced their own genome used that data for all or some analyses, what self-defined important results they generated in their analyses and with whom they discussed their results. No significant differences were observed between years for these measures. All respondents used their genomic data for all analyses, with most respondents (30, 88.2%) reporting that they received results that were important to them. A small number of students (4, 11.8%) indicated that they incorporated exclusions into their analyses.

**Table 2:** Self-reported “important” genomic results identified by students who analyzed their own genome, and with whom students discussed their results for 2014–2015 at T4, post-course (questions were not included in 2013 T4 questionnaire)

Use your genome for all analyses	T4
All	34
Some	0
Exclude regions	T4^a^
No	30
Yes	4
Receive any results felt were important	T4^b^
Yes	30
No	2
Not sure	2
If yes, in which categories	
Carrier status	18 (56%)
Pharmacogenomic	12 (38%)
Monogenic disease	15 (47%)
Physical traits	6 (19%)
Polygenic disease risk	9 (28%)
Ancestry	13 (41%)
Variant(s) of unknown significance	10 (31%)
Other	0
Discuss results with anyone	T4^c^
Yes	29
No	4
Choose not to answer	1
If yes, whom (check all that apply)	
Genetic counselor	5 (17%)
Physician or other health professional	4 (14%)
Mother	18 (62%)
Father	15 (52%)
Sibling	12 (41%)
Other family	6 (21%)
Friends	24 (83%)
Significant other	17 (59%)
Instructors	10 (34%)
Other	0
Course have impact on your family	T4^d^
Yes^e^	8
No	24
Not sure	2

^a^Chi-square test of association with year was not significant: χ^2^ (1) = 0.016, *p* = 0.90

^b^Chi-square test of association with year was not significant: χ^2^ (2) = 1.89, *p* = 0.39

^c^Chi-square test of association with year was not significant: χ^2^ (2) = 0.92, *p* = 0.63

^d^Chi-square test of association with year was not significant: χ^2^ (2) = 2.56, *p* = 0.28

^e^Free text responses to how course impacted family listed in Additional file 1: Table S6

#### Test-related distress and psychological wellbeing

**Fig. 3:**
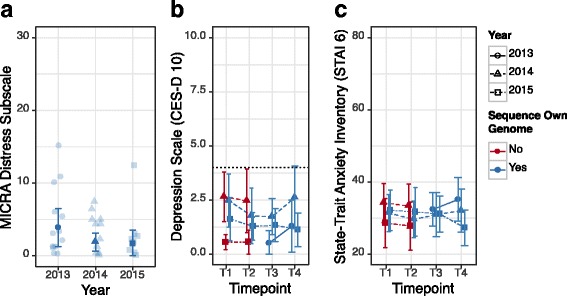
Test-related distress and psychological wellbeing by time point and year. Mean and 95% confidence interval for: **a** test-related distress (MICRA Distress subscale) post-course (T4), **b** depression (CES-D 10 Dichotomous), and **c** anxiety (STAI 6) across time point and course year. Individual scores on the MICRA Distress Subscale are shown in reduced opacity. For context, panels **b** and **c** also include reported depression and anxiety at T1 and T2 of students who did not sequence their own genome (either by choice or because they were ineligible). Since so few students at T3 and T4 could not or did not sequence their own genome, their data is not shown. The dotted line in panel **b** shows the cutoff for clinically significant depressive symptoms on the CES-D 10 Dichotomous (≥ 4) [48]

Figure 3a shows test-related distress (MICRA Distress subscale) after PAPG (T4) for students who chose to sequence their own genome. Mean (SD) MICRA Distress subscale across all years was 2.45 (3.59) and did not significantly differ between years (H(2) = 3.35, *p* = 0.19, *n* = 43). There was not a significant relationship between DCS at T2 and test-related distress at T4 (τ = 0.15, *p* = 0.21 *n* = 42).

Figure 3b and c shows measures of depression (CES-D 10 Dichotomous) and anxiety (STAI-6) across time points by course year for students who chose to sequence their own genome. For context, depression and anxiety measures at T1 and T2 for students who did not sequence their own genome are also shown. The majority of these students were enrolled only in the prerequisite course, and a minority were enrolled in PAPG but ineligible to sequence their own genome or were eligible but chose not to sequence their own genome. No significant changes were observed between T3 and T4 for either the depression (Z = 0.90, *p* = 0.37, *r* = 0.094 *n* = 46) or anxiety measures (Z = 0.26, *p* = 0.80, *r* = 0.027 *n* = 47). There was not a significant relationship between DCS at T2 and depression at T4 (τ = −0.0026, *p* = 0.98 *n* = 43) or anxiety at T4 (τ = −0.064, *p* = 0.56 *n* = 42).

#### Utility of PGS and perceived impact on student academic engagement

Tables 3, 4 and 5 summarize students’ perception of the utility of analyzing their own genome and the amount of analyses students performed outside of course assignments. Perception of utility was high at baseline and did not significantly change over the course timeline. Students who sequenced their own genome on average agreed with the proposed educational benefits of analyzing their own genome, particularly that they better understood the patient experience and that they performed greater number of and more thorough analyses. Almost all students reported analyzing additional variants outside of course assignments, including those students that did not analyze their own genome. For example, as shown in Table 5 outside of course assignments, the median student reported analyzing 6–10 additional variants and reported spending an additional 2–5 h analyzing their own genome.

**Table 3:** Mean (standard deviation) and range of Likert-scaled agreement with utility of analyzing your own genome (1-strongly disagree to 5-strongly agree)

I think analyzing my own genome would be/was useful					
	T1 (*n* = 99)	T2 (*n* = 86)	T3 (*n* = 52)	T4 (*n* = 49)	Test (*n* = 47)^a^
Eligible to sequence own genome	4.39 (0.89) 1–5	4.20 (1.05) 1–5	4.44 (0.89) 1–5	4.61 (0.76) 1–5	Z = 1.78, *r* = 0.18 *p* = 0.80
Not eligible to sequence own genome	4.00 (1.02)^b^ 1–5	3.97 (0.97)^b^ 1–5	N/A	N/A	

^a^ Wilcoxon-signed rank test between T4 and T1

^b^ 45 students not eligible to sequence own genome responded at T1, and 36 at T2

**Table 4:** Mean (standard deviation) and range of Likert-scaled agreement with engagement measures (1-strongly disagree to 5-strongly agree)

Because I used my genome^a^	T4 (*n* = 34)^b^
I was more persistent in completing assignments or analyses	4.24 (1.02) 1–5
I better understand the patient experience	4.35 (0.81) 2–5
I learned useful health or personal information	4.09 (0.93) 2–5
I better understand genetics concepts	3.94 (0.86) 2–5
I performed more analyses outside of class	4.35 (0.81) 2–5
I was more thorough in my analyses	4.35 (0.92) 2–5

^a^ Questions not in 2013 questionnaire

^b^ Wilcoxon-Mann-Whitney test of association with year was not significant for any statement

**Table 5:** The distribution of number of variants and the time students spent analyzing variants outside of course assignments

Variants analyzed outside of assignments^a^	Own Genome^b^	Ref. Genome	Hours spent analyzing genome outside of assignments^a^	Own Genome^c^	Ref. Genome
0	1	1	Less than 1	3	2
1–2	3	0	1–2	2	0
3–5	10	1	2–5	14	1
6–10	11	1	5–10	13	0
11–20	9	0	10–20	4	0
21–30	3	0	20–30	1	0
More than 30	1	0	More than 30	0	0

^a^ Questions not in 2013 questionnaire

^b^ Linear-by-linear association with year was not significant: Z = −0.49, *p* = 0.63

^c^ Linear-by-linear association with year was not significant: Z = −0.62, *p* = 0.53

### Impact of courses on genomics knowledge and application of learning

Table 6 summarizes self-reported application of knowledge gained in class in their research, clinical or other professional activities as of the end of PAPG (T4). Most students (83%) reported already applying what they learned in class.

**Table 6:** Application of knowledge gained in class from 2014 to 2015 reported at T4, post-course

Applied the knowledge gained in class	Students^a^
Yes	31
No	5
Not sure	1
If yes, in what ways?	
Using online databases	26
Computing skills	9
Variant interpretation	25
Communicating NGS capabilities and limitations	18
Genome analysis pipeline	9
Other^b^	3

^a^Chi-square test of association with year was not significant: χ^2^ (2) = 0.83, *p* = 0.66

^b^ “Other” responses listed in Additional file 1: Table S7

Table 7 summarizes self-assessed and objectively measured knowledge over the course timeline. The entire course sequence (IHGS and PAPG) had a medium-to-large positive effect on self-assessed understanding of WGS (Z = 3.22, *p* = 0.0015, *r* = 0.38, *n* = 35) and a large and significant positive effect on objectively measured knowledge (Z = 4.40, *p* = 0.24e-7, *r* = 0.57, *n* = 30). Since almost all PAPG students have chosen to sequence their own genome, we cannot meaningfully compare changes between those who did and did not sequence their own genome. There was not a significant relationship between test-related distress reported at T4 and objective knowledge at T4 (τ = 0.16, *p* = 0.30, *n* = 28) or the change in objective knowledge over PAPG (τ = −0.0040, *p* = 0.98, *n* = 26) for students who chose to sequence their own genome.

**Table 7:** Mean (standard deviation) and range of self- and objectively-assessed genetics and genomics knowledge across all students and time points

Self-assessed knowledge	T1 (*n* = 65)	T2 (*n* = 57)	T3 (*n* = 52)	T4 (*n* = 52)	Test (*n* = 35)^a^
Confidence	2.9 (1.1) 1–5	3.4 (0.9) 1–5	3.1 (1.0 1–5	3.6 (0.7)^b^ 1–5	Z = 2.82, *r* = 0.37 *p* = 0.0041
Current understanding of genetics	4.17 (0.78) 2–5	4.09 (0.71) 2–5	4.31 (0.64) 3–5	4.19 (0.69) 3–5	Z = −0.30, *r* = 0.04 *p* = 0.99
Genetics knowledge compared to others	4.11 (0.89) 2–5	4.05 (0.66) 2–5	4.19 (0.66) 2–5	4.46 (0.58) 3–5	Z = 2.01 *r* = 0.24 *p* = 0.065
Current understanding of WGS	3.68 (0.87) 2–5	3.88 (0.71) 2–5	3.75 (0.74) 2–5	4.08 (0.65) 3–5	Z = 3.22, *r* = 0.38 *p* = 0.0015
Current WGS knowledge compared to others	3.86 (0.83) 2–5	3.95 (0.85) 2–5	3.92 (0.79) 2–5	4.42 (0.54) 3–5	Z = 3.13, *r* = 0.37 *p* = 0.0026
Objective knowledge	T1 (*n* = 60)	T2 (*n* = 53)	T3 (*n* = 37)	T4 (*n* = 33)	Test (*n* = 30)^a^
Genomics test	2.5 (1.7)^**c**^ 0–7	3.1 (1.5) 0–7	2.9 (1.7) 0–7	4.3 (1.8)^**d**^ 1–9	Z = 4.40, *r* = 0.57 *p* = 9.24e-7

Confidence is reported on a 1–5 scale from “No confidence” to “High confidence”. Current understanding is reported on a 1–5 scale from “None” to “High”, while knowledge compared is reported on a 1–5 scale from “Much less than others” to “Much more than others”. Test results are reported for paired comparison of T1 and T4. Total number of valid responses shown at each time point. The objective knowledge measure, range 0–10, was only included in 2014–2015

^a^Wilcoxon-signed rank test between T4 and T1, and thus only including eligible students

^b^Only 44 participants answered this question at T4

^c^Wilcoxon-Mann-Whitney test of association with year was not significant: Z = −1.11, *p* = 0.27

^d^ Wilcoxon-Mann-Whitney test of association with year was not significant: Z = −1.27, *p* = 0.21

### Impact of course sequence on attitudes toward genome sequencing

In general students’ responses to the attitudes statements did not change significantly over the course sequence (T4 vs. T1), with the exception of increased agreement with the statements “I know enough about genetics to understand the whole genome sequencing results” (Z = 3.53, *p* = 3.4e-4, *r* = 0.35, *n* = 50) and “I understand the risks and benefits of using/getting personal whole genome sequencing done” (Z = 3.88, *p* = 6e-5, *r* = 0.39, *n* = 50), and increased disagreement with “Concerns about privacy/risks to privacy” is a reason to not use your own genome in class (Z = −3.66, *p* = 1.6e-4, r-0.44, *n* = 34). Additional file 1: Tables S8-S10 summarize responses to the attitude measures. Responses to the above statements at T4 did not significantly differ between years: “I know enough…” (H(2) = 5.52, *p* = 0.063, *n* = 52); “I understand the…” (H(2) = 5.28, *p* = 0.07, *n* = 52); and “Concerns about privacy…” (Z = 2.21, *p* = 0.027, *n* = 36).

## Discussion

The results reported here from the 2013–2015 PAPG cohorts reinforce and extend our observations from the initial 2012 cohort [16, 17]: students’ interest in sequencing their own genome was high, students felt their decision was more informed after the prerequisite workshop, and, at the completion of PAPG, most, but not all, students who sequenced their own genome reported low levels of decision regret and test-related distress. To the instructors and investigators knowledge, no 2013–2015 students experienced a focused episode of test-related distress like that reported by a 2012 student in response to a variant of unknown significance in a gene associated with adult-onset incompletely penetrant monogenic disease [17].

Although personal genomes play a large role in PAPG, homework and other course exercises are necessarily designed around instructor-provided genomic data so that there is a known correct answer and so that students aren’t required to “turn in” their own genomic data. Thus in many respects students have a typical pedagogical experience that includes the supervised, team-based, genomic analysis they will later perform professionally [30]. But they are then further exercising those new skills analyzing their own genomes (albeit more autonomously than would be the case in a clinical setting). Students reported also performing most or all of the same analyses on their own genomes, with a majority of students reporting spending 2–5 or 5–10 additional hours analyzing their genome outside of course exercises and assignments. And most, but not all, students agreed or strongly agreed that they were more persistent and performed more, and more thorough, analyses because they analyzed their own genomes. These quantitative data extends the pilot qualitative results from the 2012 PAPG cohort [17]. And these results are consistent with reports from other courses incorporating personal genomic testing (typically genotyping) in which students generally report that personal genomic testing enhanced their learning and improved their understanding of genomic testing and the patient experience [31–39].

We cannot, however, quantitatively determine whether incorporating PGS improves educational outcomes. Both self-assessed and objective measures of genomics knowledge increase after completing PAPG. However, since almost all students have elected to sequence their own genome, there is an insufficient comparison group to meaningfully evaluate the differences between students who did and did not sequence their own genome. And the context of PAPG did not allow the study to be implemented as a controlled randomized trial that could assess causality. Additional data will be needed to quantitatively assess the pedagogical value of PGS in PAPG.

The prerequisite workshop is intended to both educate and promote informed decision-making. As indicated by the significant decrease in decisional conflict, students feel that their decision was more informed after completing the prerequisite workshop. And afterwards fewer students were concerned that they would be at a disadvantage if they did not sequence their own genome and none of the respondents agreed that they were concerned that the faculty would know their choice. The educational setting creates additional potential for coercion; students could feel pressured to sequence their own genome, directly or indirectly, by the faculty or their peers. Ensuring that students do not feel disadvantaged in choosing a reference genome and do feel that their choice is private are both important to mitigating the possibility of coercion. These results indicate that the course design and sequencing protocol are achieving the above, and that the instructors are effectively communicating the protections that are in place.

We note students’ decisional conflict is higher both at baseline and after the prerequisite workshop than that reported by participants in HealthSeq pre-dispositional PGS study (mean of 11.34 before and 5.94 after genomic counseling) [40]. This difference could reflect the many differences between these cohorts, including that the HealthSeq cohort is self-selected (individuals would likely not enroll in that study if they did not want genetic findings from WGS) whereas approximately half of PAPG students are required to enroll in the course, or that PAPG students are more knowledgeable about WGS than the general public. As more individuals obtain predispositional PGS [18] we will develop a better understanding of the correlates of informed decision-making; that data will inform development of much-needed best practices for genomic counseling of ostensibly healthy individuals.

We observed student attitudes and outcomes to be similar between course years, except at baseline. Baseline interest increased and decisional conflict decreased each year, that is students increasingly began the course with more certainty in their decision to sequence their own genome or not. Over the study period the course became more established and students had one or more years to anticipate enrollment and the decision to sequence their own genome. Concurrently genome sequencing became more widely used in both clinical and research settings and thus students are more likely to have already encountered this technology at baseline. Thus we hypothesize that over time more of the decision-making process takes place prior to enrolling in the PAPG course sequence. And indeed we observed that in 2014 and 2015 baseline mean decisional conflict was below the threshold associated with implementing a decision, and baseline average agreement with both “I know enough about genetics to understand the WGS results” and particularly “I understand the risks and benefits of using/getting personal WGS done” increased each year. These results suggest that courses like PAPG, and perhaps predispositional PGS more generally, may need to be evaluated over several years to determine “steady-state” measures of decision-making.

Limitations for this study include: the single site; a larger but still small sample size with missing responses; and the possibility for students under- or over-reporting variables that may be critical or supportive of the course design, respectively, out of concern for impacts on the institution, instructors and the continued availability of the course. The online questionnaires incorporated themes from follow-up qualitative interviews with initial 2012 cohort [17], but necessarily would not have captured all attitudes or outcomes that may have been elicited in an interview. With a predicted rate of health-relevant adult-onset monogenic disease variants on the order of 1–5% [41–43], this study is not yet well powered to detect distress, and impacts thereof, caused by these rare variants. And since all impacts are self-reported, students may have experienced test-related distress but not reported it on the questionnaires or to the course or study team. Where possible we utilized validated and widely used measures that permit comparison to other cohorts and contexts. At present, however, there is no common, rigorously validated, measure of genomics knowledge making it difficult to compare PAPG to other educational approaches [44]. Developing such a measure is a key area of future work. PAPG is a required course for approximately half of the students, cohorts that are entirely self-selected (or entirely required) may respond differently to PGS in an educational setting.

## Conclusions

PAPG is one of several experiments in participatory genomics pedagogy [44] that also include incorporating personal genotyping [31–39, 45], analyzing cadaver genomes in anatomy lab [46], and bench-top sequencing [47]. At present, the cost and complexity of WGS are barriers to implementing educational PGS more widely. However, in the future we expect WGS to be widely available at low cost; thus the question of whether to incorporate PGS into genomics education will be strictly of the balance between the educational benefits and the possible adverse effects [11]. Here we showed that: the prerequisite workshop and associated materials promoted more informed decision-making about PGS; most, but not all students, reported low levels of decision regret and test-related distress; and students reported being more engaged and persistent as a result of sequencing and analyzing their own genome as part of the course. We hope this report about our multi-year experience incorporating PGS into graduate-level genomics education will contribute to the important ongoing discussion on how to most effectively train the much-needed next-generation of genomics professionals.

## Additional files

Additional file 1: Supplemental methods and data referenced in text. (PDF 183 kb)

Additional file 2: Questionnaire administered at time points T1 and T2. (PDF 680 kb)

Additional file 3: Questionnaire administered at time point T3. (PDF 725 kb)

Additional file 4: Questionnaire administered at time point T4. (PDF 791 kb)

## Abbreviations

PGS: Personal genome sequencing
WGS: Whole genome sequencing
