# Supplementary material for: Impacts of incorporating personal genome sequencing into graduate genomics education: a longitudinal study over three course years
Source: BMC Med Genomics. 2018 Jan 30;11:5. doi: 10.1186/s12920-018-0319-0 (PMC5791365; doi:10.1186/s12920-018-0319-0)
Supplement: Supplementary file 1 — Supplemental methods and data referenced in text. (PDF 183 kb) [file 12920_2018_319_MOESM1_ESM.pdf]

Impacts of Incorporating Personal Genome Sequencing into Graduate Genomics Education: A Longitudinal Study Over Three Course Years

Supplemental Methods and Data

Michael D. Linderman[1,2,5], Saskia C. Sanderson[1,2,3,4], Ali Bashir[1,2], George A. Diaz[2], Andrew Kasarskis[1,2], Randi Zinberg[2], Milind Mahajan[1,2], Sabrina A. Suckiel[1,2], Micol Zweig[1,2], Eric E. Schadt[1,2]

[1]Icahn Institute for Genomics and Multiscale Biology, Icahn School of Medicine at Mount Sinai, New York, NY

[2]Department of Genetics and Genomic Sciences, Icahn School of Medicine at Mount Sinai, New York, NY

[3]Health Behaviour Research Centre, Department of Epidemiology and Public Health, University College London, UK

[4]Department of Clinical Genetics, Great Ormond Street Hospital, London, UK

[5]Department of Computer Science, Middlebury College, Middlebury, VT, USA

Supplemental Methods

Study and Course Design

The study protocol, course design, course sequencing protocol and approval process for the both the course and study are described in more detail in related publications[1–3]. The course and study timeline are summarized in Figure S1.

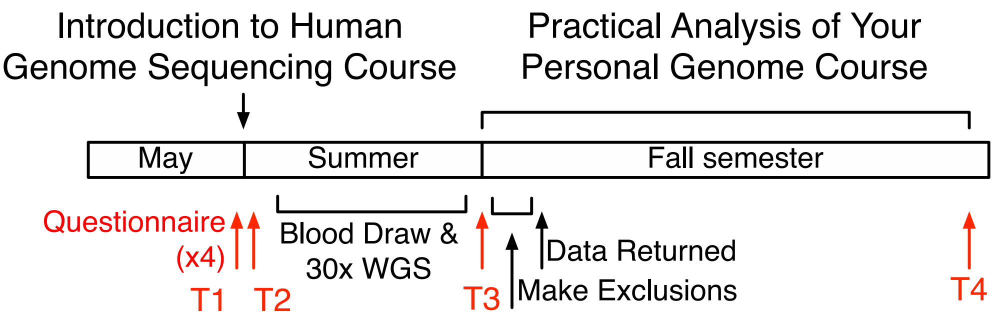

Figure S1: Course and study timeline show questionnaires, decision-making, sequencing and data return. Adapted from Linderman et al[3].

Measures

Table S1 and Table S2 list the measures administered in each questionnaire. The measures and any modifications are briefly described below and in more detail in prior publications[1,2].

Decisional Conflict: Decisional conflict was assessed with 16-item Decisional Conflict Scale (DCS)[4,5] as described in the user manual[5]. DCS item 10 was not included in the questionnaire and so was mean imputed from the remaining 15 items[1]. The total DCS has a range of 0-100; a score < 25 is associated with implementing a decision and a score > 37.5 is associated with feeling unsure about a decision.

Satisfaction with Decision: Satisfaction with decision was measured with the Satisfaction with Decision Scale (SWD)[6]. The introductory text was adapted to the context of WGS decision-making. The total scale has a range of 1-5 with 1 indicating low satisfaction and 5 indicating high satisfaction with the decision. In a previous study of women's decisions regarding management of menopause and hormone replacement therapy, the mean (SD) SWD score 3.9 (0.60)[6].

Decision Regret: Decision regret was measure with the Decision Regret Scale (DRS)[7]. The total scale ranges from 0-100 with higher values indicating increased regret. Previously reported mean scores ranged from 8.5 to 25.4 among various patient cohorts who had made healthcare-related decisions[7].

Anxiety: Anxiety was assessed with the short form of the State-Trait Anxiety Inventory (STAI-6)[8]. For the 2013 questionnaires, STAI-6 scores were derived from 20-question STAI-20 using items 1,3,6,15,16, and 17[9]. The STAI-6 scores were scaled to the same 20-80 range as the STAI-20.

Depression: Depression was assessed with the 10-question dichotomous Center for Epidemiologic Studies Depression Scale (CES-D 10)[10]. For the 2013 questionnaire CES-D 10 scores were derived from the longer CES-D 20[11] using items 6,7,11,12,14,15,16,18,19, and 20 and mapping responses of “Much of the time” and “Most or all of the time” to “Yes. CES-D 10 has a range of 0-10; a score of ≥ 4 is associated with clinically significant depressive symptoms[12].

Test-related Distress: Test-related distress was assessed with a modified version of the Multidimensional Impact of Cancer Risk Assessment (MICRA)[13]. We administered the 21 core items plus the items for respondents with children. As described previously, we adapted the measure items to be more relevant to WGS[2]. The MICRA Distress subscale comprises 6-items with total range of 0-30.

Objective Knowledge: Objective knowledge was assessed with a newly developed 10-question multiple-choice test. All correct answers are required for the question to be considered correct and scored as 1. The “Don’t know” option was scored as 0, the same as an incorrect response.

Question	Correct answers
1	3 and 7
2	4 and 5
3	2
4	1 (3 and 4 were ignored as potentially ambiguous)
5	2 and 4
6	3
7	3
8	4 (1 was ignored as potentially ambiguous)
9	1 and 3
10	5

Table S1: Measures in 2013 questionnaires at each time point.

Measure	Source	T1	T2	T3	T4
Interest in analyzing own genome	O'Connor 1995	X	X	X	
Decisional Conflict	O'Connor 1995	X	X	X	
Perceived utility of WGS in class	Ormond 2011	X	X	X	
Attitudes re: personal WGS in class	Ormond 2011	X	X	X	X
Attitudes re: WGS generally	Ormond 2011	X	X	X	X
Intention to use own genome	New			X	
Discussed WGS decision with others	New			X	
Actual WGS decision	New				X
Decision Regret	Brehaut 2012				X
Decision Satisfaction	Holmes-Rovner 1995				X
Actual utility	Ormond 2011				X
Analyses actually performed	New				
WGS results obtained	New				
Discussed WGS results	New				X
Psychological impact of WGS results	MICRA				X
Impact of course on family	New				
Impact of course on professional practice	New				
Anxiety	STAI-20			X	X
Depression	CES-D 20			X	X
Subjective understanding of genetics/genomics	MSSM Healthy Subjects			X	X
Confidence in ability to analyze WGS	New			X	X
Objective knowledge about WGS	Sanderson 2013	X	X	X	X

Table S2: Measures in 2014 and 2015 questionnaires at each time point. 'NS' questionnaires were sent to genome ineligible students, i.e. students enrolled in PAPG without the option to sequence their own genome.

Measure	Source	T1	T2	T3	T3 NS	T4	T4 NS
Interest in analyzing own genome	O'Connor 1995	X	X		X		
Decisional Conflict	O'Connor 1995	X	X	X	X		
Perceived utility of WGS in class	Ormond 2011	X	X	X	X		
Attitudes re: personal WGS in class	Ormond 2011	X	X	X	X	X	X
Attitudes re: WGS generally	Ormond 2011	X	X	X	X	X	X
Discussed WGS decision with others	New			X			
Actual WGS decision	New			X		X	
Intentions for analysis	New			X			
Decision Regret	Brehaut 2012					X	
Decision Satisfaction	Holmes-Rovner 1995			X		X	
Actual utility	Ormond 2011					X	
Analyses actually performed	New					X	
WGS results obtained	New					X	
Discussed WGS results	New/PeopleSeq					X	
Psychological impact of WGS results	MICRA					X	
Impact of course on family	New					X	X
Impact of course on professional practice	New					X	X
Anxiety	STAI-6	X	X	X	X	X	X
Depression	CES-D 10 Dichotomous	X	X	X	X	X	X
Engagement	New					X	X
Subjective understanding of genetics/genomics	MSSM Healthy Subjects	X	X	X	X	X	X
Confidence in ability to analyze WGS	New	X	X	X	X	X	X
Objective knowledge about WGS	New	X	X	X	X	X	X

Supplemental Data

Table S3: Student enrollment during the study period. The numbers of PAPG students enrolled without the option to sequence their own genome are shown in parentheses. "Other" includes practicing genetic counselors, post-doctoral fellows and nurses.

Student Program or Specialty	2013		2014		2015	
	IHGS Only	IHGS + PAPG	IHGS Only	IHGS + PAPG	IHGS Only	IHGS + PAPG
Genetic Counseling Students	9	8	8	8	12	12
Graduate Students (incl. MD/PhD)	11	9[1]	16	13(5)[2]	10	8(2)[3]
Medical Genetics Residents	1	1	3	3	1	1
Laboratory Geneticists and Molecular Pathologists	4	1	6	1	5	1
Faculty/Attending	3	0	0	0	4	0
Other	10	0	2	0	4	0

[1] Two of these students dropped the course during the semester

[2] Two of the students who enrolled without the option to obtain their genome dropped the course during the semester.

[3] One of the students who enrolled without the option to obtain their genome dropped the course during the semester.

Table S4: Mean (standard deviation) and range of Decisional Conflict Scale (DCS), including subscales, for genome eligible students in all course years.

DCS Subscale	T1 (n=56)	T2 (n=57)	Test (n=51)[1]
Total Scale	24.29 (15.86)<br>0-60	15.80 (13.60)<br>0-50	Z=-3.76, r=0.37<br>*p*=0.000093
Uncertainty Subscale	26.25 (20.61)<br>0-70	21.61 (20.10)<br>0-67	Z=-1.83, r=0.18<br>*p*=0.066827
Informed Subscale	23.83 (18.40)<br>0-75	10.74 (11.82)<br>0-33	Z=-4.16, r=0.41<br>*p*=0.000011
Values Subscale	29.24 (21.77)<br>0-83	18.24 (16.99)<br>0-67	Z=-3.74, r=0.36<br>*p*=0.000098
Support Subscale	19.88 (17.94)<br>0-67	12.11 (12.72)<br>0-58	Z=-2.44, r=0.24<br>*p*=0.013644
Effective Subscale	21.76 (16.64)<br>0-69	15.45 (16.10)<br>0-50	Z=-3.30, r=0.32<br>*p*=0.000691

[1] Wilcoxon-signed rank test

Table S5: Mean (standard deviation) and range for Likert-scale agreement (1=Strongly Disagree, 5=Strongly Agree) with potential benefits and concerns for sequencing and analyzing your own genome for genome eligible students 2013-2015.

Benefit or Concern	T1 (n=56)	T2 (n=52)	Test (n=52)[1]
My own results would help me understand genetics concepts better than someone else's results	3.72 (1.13) 1-5	3.70 (1.19) 1-5	Z=-0.38, r=0.04 p=0.80
I feel that I would be at a disadvantage to my classmates if I did not undergo the testing	2.50 (0.87) 1-4	2.00 (0.97) 1-5	Z=-3.35, r=0.33 p=0.00073
I would see this as an opportunity to get a service that I would not ordinarily get if I had to pay full price	4.40 (0.82) 2-5	4.38 (0.66) 2-5	Z=-0.82, r=0.08 p=0.48
I would be concerned that my professors would know who took up the offer of testing and who did not	1.75 (0.66) 1-3	1.53 (0.54) 1-3	Z=-2.38, r=0.23 p=0.022
I would be concerned that my classmates would know who took up the offer of testing and who did not	1.79 (0.70) 1-3	1.70 (0.67) 1-4	Z=-0.62, r=0.06 p=0.54
I would see this as an opportunity to get information that would help me improve my health	4.04 (0.80) 1-5	4.06 (0.72) 2-5	Z=0.21, r=0.02 p=1.00
I would be concerned that I might get some results that would be disturbing	3.65 (0.77) 2-5	3.65 (0.86) 2-5	Z=0.00, r=0.00 p=1.00
I would only take up the offer of testing if I could get genetic counseling before I sent my sample in	2.44 (0.91) 1-4	2.66 (0.98) 1-5	Z=2.05, r=0.20 p=0.05
I would only take up the offer of testing if I could get genetic counseling after I got my results back in.	2.95 (1.09) 1-5	3.13 (1.04) 1-5	Z=1.33, r=0.13 p=0.19
I would be concerned that people would find out genetic or health information about me.	2.74 (1.09) 1-5	2.40 (0.88) 1-4	Z=-1.80, r=0.17 p=0.071
I would only take up the offer of testing if I could exclude parts of the genome that I did not want to look at.	2.58 (1.18) 1-5	2.75 (1.02) 1-5	Z=0.74, r=0.07 p=0.47

[1] Wilcoxon-signed rank test

Table S6: Free text responses to how course had an impact on respondents' family at T4. Question was not included in 2013 T4 questionnaire.

Year	If the course had an impact on your family, how?
2014	"Told my father about our Alzheimer's risk, which is the same as the general population for ApoE. We still have Alzheimer's in the family and therefore a higher risk, but it was still relieving to see that that locus was not involved."<br><br>"My mother became extremely interested in the ancestry analysis, so much so that she ordered a 23andme kit to find out to explore her own."<br><br>"I gave them some information about risks"
2015	"I think they are more knowledgeable and maybe curious."<br><br>"Mother is much more concerned about a finding that I had and used it as an opportunity to bring up her hate for my father!"<br><br>"They are thinking about it more than they were before."<br><br>"I told my brother that I was a carrier for classical galactosemia which gave him anxiety because he and his wife are expecting a baby."<br><br>"I found I was a carrier for something and shared the information with my family."<br><br>"Tell my siblings they need carrier screening when reach childbearing age, prompt to check on EKG results."

Table S7: "Other" impact on professional practice free text responses at T4. Question was not included in 2013 T4 questionnaire.

Year	Other impact on professional practice free text responses
2014	"Ion Torrent cancer hotspot data analysis, interpretation, and improvement"
2015	"Risk prediction for disease based on genomics, i.e. calculating polygenic load - I expect this to be very helpful in my research."<br><br>"Understanding literature and more able to discuss genetics pipelines and technical issues with others."

Table S8: Views on utility of (1=Not useful at all, 5=Very useful) and likelihood of behavioral change (1=Not all likely, 5=Very likely) in response to WGS for students who were genome eligible (E) and not genome eligible (Ē).

		T1	T2	T3	T4	Test[1]
How useful do you think the results from whole genome sequencing will be to a physician?	E	3.49 (0.83) 2-5 (n=57)	3.51 (0.87) 2-5 (n=53)	3.56 (0.75) 2-5 (n=52)	3.38 (1.11) 1-5 (n=45)	Z=-0.53, r=0.06 *p*=0.60 (n=45)
	Ē	3.67 (1.07) 1-5 (n=42)	3.62 (0.94) 1-5 (n=32)	N/A	N/A	
How useful do you think the results from whole genome sequencing will be to a patient?	E	3.53 (0.89) 2-5 (n=57)	3.58 (0.93) 2-5 (n=53)	3.71 (0.92) 1-5 (n=51)	3.52 (0.75) 2-5 (n=27)	Z=-0.35, r=0.05 *p*=0.82 (n=26)
	Ē	3.33 (1.18) 1-5 (n=42)	3.59 (1.10) 1-5 (n=32)	N/A	N/A	
How likely is it that knowing the results from whole genome sequencing for yourself would lead to any changes in your behavior?	E	2.88 (1.04) 1-5 (n=57	3.02 (1.03) 2-5 (n=53)	2.75 (1.02) 1-5 (n=53)	2.91 (1.08) 1-5 (n=34)	Z=0.30, r=0.04 *p*=0.78 (n=34)
	Ē	3.21 (0.98) 2-5 (n=42)	3.38 (1.16) 2-5 (n=32)	N/A	N/A	

[1] Wilcoxon-signed rank test T4 vs. T1

Table S9: Likert-scale agreement (1=Strongly disagree, 5=Strongly agree) with statements about reasons for and reasons against sequencing your own genome in a genomics class for students who were genome eligible (E) and not genome eligible (Ē).

		T1	T2	T3	T4	Test[1]
**Reasons for using own genome**						
Satisfy general curiosity	E	4.54 (0.71)<br>2-5 (n=57)	4.68 (0.61)<br>2-5 (n=53)	4.62 (0.59)<br>2-5 (n=55)	4.77 (0.43)<br>4-5 (n=34)	Z=0.63, r=0.08<br>p=0.75 (n=33)
	Ē	4.21 (0.87)<br>2-5 (n=42)	4.28 (0.63)<br>2-5 (n=32)	N/A	N/A	
See if a specific disease runs in the family or is in DNA	E	4.00 (1.04)<br>1-5 (n=41)	3.91 (1.01)<br>1-5 (n=32)	4.00 (0.85)<br>2-5 (n=55)	4.40 (0.69)<br>3-5 (n=35)	Z=1.84, r=0.22<br>p=0.070 (n=34)
	Ē	4.02 (1.01)<br>1-5 (n=56)	4.00 (0.89)<br>2-5 (n=53)	N/A	N/A	
Learn about genetic makeup without going through a physician	E	3.70 (1.02)<br>1-5 (n=57)	3.66 (1.16)<br>1-5 (n=53)	3.82 (1.09)<br>1-5 (n=55)	3.89 (1.21)<br>1-5 (n=35)	Z=0.36, r=0.04<br>p=0.73 (n=35)
	Ē	3.74 (1.11)<br>1-5 (n=42)	3.84 (0.88)<br>2-5 (n=32)	N/A	N/A	
Inform family members about health risks	E	3.48 (1.04)<br>1-5 (n=56)	3.42 (0.89)<br>2-5 (n=53)	3.46 (1.08)<br>1-5 (n=54)	3.94 (1.03)<br>1-5 (n=35)	Z=0.73, r=0.09<br>p=0.52 (n=35)
	Ē	3.95 (0.93)<br>2-5 (n=38)	3.70 (1.06)<br>1-5 (n=30)	N/A	N/A	
Understand what a patient may learn/experience	E	4.32 (0.81)<br>2-5 (n=57)	4.23 (0.88)<br>2-5 (n=52)	4.25 (0.84)<br>2-5 (n=55)	4.29 (0.89)<br>1-5 (n=35)	Z=-0.68, r=0.08<br>p=0.52 (n=35)
	Ē	4.17 (0.67)<br>2-5 (n=41)	4.16 (0.69)<br>2-5 (n=31)	N/A	N/A	
Help understand principles of human genetics	E	4.36 (0.82)<br>2-5 (n=55)	4.15 (0.84)<br>2-5 (n=53)	4.31 (0.91)<br>2-5 (n=54)	4.34 (0.87)<br>2-5 (n=35)	Z=-0.85, r=0.10<br>p=0.47 (n=34)
	Ē	4.00 (0.97)<br>1-5 (n=41)	4.25 (0.72)<br>2-5 (n=32)	N/A	N/A	
**Reasons against using own genome**						
Results are not reliable	E	2.58 (0.75)<br>1-4 (n=53)	2.68 (0.91)<br>1-4 (n=50)	2.55 (0.87)<br>1-5 (n=49)	2.18 (0.80)<br>1-4 (n=34)	Z=-2.89, r=0.36<br>p=0.0059 (n=33)
	Ē	2.39 (0.80)<br>1-4 (n=41)	2.52 (0.93)<br>1-4 (n=31)	N/A	N/A	
Results are not accurate	E	2.47 (0.80)<br>1-4 (n=53)	2.60 (0.88)<br>1-4 (n=50)	2.53 (0.92)<br>1-5 (n=49)	2.15 (0.82)<br>1-4 (n=34)	Z=-2.42, r=0.30<br>p=0.019 (n=33)
	Ē	2.39 (0.74)<br>1-4 (n=41)	2.55 (0.93)<br>1-4 (n=31)	N/A	N/A	
Results are not predictive	E	3.08 (0.85)<br>1-4 (n=53)	3.04 (0.92)<br>1-5 (n=50)	3.00 (0.92)<br>1-5 (n=48)	2.88 (1.09)<br>1-5 (n=34)	Z=-1.52, r=0.19<br>p=0.16 (n=33)
	Ē	2.58 (0.87)<br>1-5 (n=40)	2.94 (0.96)<br>1-4 (n=31)	N/A	N/A	
Concern about privacy/risks to privacy	E	3.07 (1.24)<br>1-5 (n=54)	2.42 (0.94)<br>1-4 (n=36)	2.61 (1.24)<br>1-5 (n=49)	2.15 (1.10)<br>1-4 (n=34)	Z=-3.66, r=0.44<br>p=1.7e-4 (n=34)
	Ē	3.44 (1.16)<br>2-5 (n=41)	3.42 (0.96)<br>2-5 (n=32)	N/A	N/A	
Information will not be medically useful/will not change medical decisions	E	2.53 (1.01)<br>1-5 (n=53)	2.74 (0.83)<br>1-4 (n=50)	2.43 (0.89)<br>1-4 (n=49)	2.38 (1.02)<br>1-4 (n=34)	Z=-0.85, r=0.10<br>p=0.42 (n=33)
	Ē	2.98 (1.12)<br>1-5 (n=40)	2.87 (1.12)<br>1-5 (n=31)	N/A	N/A	
Information will not help learn human genetics	E	1.85 (0.93)<br>1-5 (n=53)	1.74 (0.75)<br>1-4 (n=50)	1.88 (0.83)<br>1-4 (n=49)	1.68 (0.64)<br>1-4 (n=34)	Z=0.52, r=0.06<br>p=0.63 (n=33)
	Ē	2.07 (0.69)<br>1-4 (n=41)	2.13 (0.90)<br>1-4 (n=30)	N/A	N/A	
Unwanted information	E	2.72 (1.28)<br>1-5 (n=53)	2.88 (1.27)<br>1-5 (n=50)	2.90 (1.28)<br>1-5 (n=49)	2.59 (1.40)<br>1-5 (n=34)	Z=-0.31, r=0.04<br>p=0.77 (n=33)
	Ē	3.05 (1.20)<br>1-5 (n=41)	3.13 (1.26)<br>1-5 (n=31)	N/A	N/A	

[1] Wilcoxon-signed rank test T4 vs. T1

Table S10: Likert-scale agreement (1=Strongly disagree, 5=Strongly agree) with statements about WGS for students who were genome eligible (E) and not genome eligible (Ē).

		T1	T2	T3	T4	Test[1]
**Please respond to the follow statements**						
Whole genome sequencing is useful for patients.	E	3.88 (0.68) 2-5 (n=57)	3.72 (0.72) 2-5 (n=53)	3.82 (0.67) 2-5 (n=55)	3.84 (0.79) 2-5 (n=50)	Z=0.46, r=0.05 p=0.68 (n=50)
	Ē	3.60 (0.91) 1-5 (n=42)	3.62 (0.98) 1-5 (n=32)	N/A	N/A	
Physicians have a professional responsibility to help individuals understand the results they receive from whole genome sequencing, even if the physician has not ordered the test.	E	3.49 (1.04) 1-5 (n=57)	3.47 (1.07) 1-5 (n=53)	3.55 (1.10) 1-5 (n=55)	3.74 (0.99) 2-5 (n=47)	Z=1.16, r=0.12 p=0.26 (n=47)
	Ē	3.29 (1.11) 1-5 (n=42)	3.09 (1.12) 1-5 (n=32)	N/A	N/A	
Physicians have enough knowledge to help individuals interpret results of whole genome sequencing.	E	1.98 (0.81) 1-4 (n=57)	2.09 (0.71) 1-4 (n=53)	2.09 (0.70) 1-3 (n=55)	2.14 (0.86) 1-5 (n=37)	Z=1.37, r=0.16 p=0.19 (n=37)
	Ē	2.26 (0.96) 1-4 (n=42)	2.25 (1.02) 1-5 (n=32)	N/A	N/A	
Most people can accurately interpret whole genome sequencing results	E	1.42 (0.53) 1-3 (n=57)	1.40 (0.49) 1-2 (n=53)	1.47 (0.54) 1-3 (n=55)	1.63 (0.69) 1-4 (n=27)	Z=0.90, r=0.12 p=0.55 (n=27)
	Ē	1.55 (0.59) 1-3 (n=42)	1.72 (0.68) 1-3 (n=32)	N/A	N/A	
I know enough about genetics to understand the whole genome sequencing results	E	2.96 (1.03) 1-5 (n=57)	3.28 (0.95) 1-5 (n=53)	2.96 (1.02) 1-5 (n=55)	3.58 (0.88) 1-5 (n=50)	Z=3.53, r=0.35 p=3.4e-4 (n=50)
	Ē	2.90 (1.03) 1-5 (n=42)	3.16 (1.14) 1-5 (n=32)	N/A	N/A	
I understand the risks and benefits of using/getting personal whole genome sequencing done	E	3.95 (0.91) 2-5 (n=57)	4.30 (0.50) 3-5 (n=53)	4.38 (0.49) 4-5 (n=55)	4.54 (0.50) 4-5 (n=50)	Z=3.88, r=0.39 p=6.0e-5 (n=50)
	Ē	3.74 (0.86) 2-5 (n=42)	3.97 (0.74) 2-5 (n=32)	N/A	N/A	

[1] Wilcoxon-signed rank test T4 vs. T1

References

1. Sanderson SC, Linderman MD, Kasarskis A, Bashir A, Diaz GA, Mahajan M, et al. Informed decision-making among students analyzing their personal genomes on a whole genome sequencing course: a longitudinal cohort study. Genome Med. 2013;5: 113. doi:10.1186/gm518
2. Sanderson SC, Linderman MD, Zinberg R, Bashir A, Kasarskis A, Zweig M, et al. How do students react to analyzing their own genomes in a whole-genome sequencing course?: outcomes of a longitudinal cohort study. Genet Med. 2015;17: 866–74. doi:10.1038/gim.2014.203
3. Linderman MD, Bashir A, Diaz GA, Kasarskis A, Sanderson SC, Zinberg RE, et al. Preparing the next generation of genomicists: a laboratory-style course in medical genomics. BMC Med Genomics. 2015;8: 47. doi:10.1186/s12920-015-0124-y
4. O'Connor AM. Validation of a decisional conflict scale. Med Decis Making. 15: 25–30.
5. O'Connor A. User Manual - Decisional Conflict Scale (16 item statement format). Ottawa; 1993.
6. Holmes-Rovner M, Kroll J, Schmitt N, Rovner DR, Breer ML, Rothert ML, et al. Patient satisfaction with health care decisions: the satisfaction with decision scale. Med Decis Making. 16: 58–64.
7. Brehaut JC, O'Connor AM, Wood TJ, Hack TF, Siminoff L, Gordon E, et al. Validation of a decision regret scale. Med Decis Making. 23: 281–92.
8. Marteau TM, Bekker H. The development of a six-item short-form of the state scale of the Spielberger State-Trait Anxiety Inventory (STAI). Br J Clin Psychol. 1992;31 (Pt 3): 301–6.
9. Spielberger CD, Gorsuch RL, Lushene R, Vagg PR, Jacobs GA. Manual for the State-Trait Anxiety Inventory. Palo Alto, CA: Consulting Psychologists Press; 1983.
10. Kohout FJ, Berkman LF, Evans DA, Cornoni-Huntley J. Two shorter forms of the CES-D (Center for Epidemiological Studies Depression) depression symptoms index. J Aging Health. 1993;5: 179–93.
11. Radloff LS. The CES-D Scale: A Self-Report Depression Scale for Research in the General Population. Appl Psychol Meas. 1977;1: 385–401. doi:10.1177/014662167700100306
12. Irwin M, Artin KH, Oxman MN. Screening for depression in the older adult: criterion validity of the 10-item Center for Epidemiological Studies Depression Scale (CES-D). Arch Intern Med. 159: 1701–4.
13. Cella D, Hughes C, Peterman A, Chang C-H, Peshkin BN, Schwartz MD, et al. A brief assessment of concerns associated with genetic testing for cancer: the Multidimensional Impact of Cancer Risk Assessment (MICRA) questionnaire. Health Psychol. 2002;21: 564–72.
