# Supplementary material for: Impacts of incorporating personal genome sequencing into graduate genomics education: a longitudinal study over three course years
Source: BMC Med Genomics. 2018 Jan 30;11:5. doi: 10.1186/s12920-018-0319-0 (PMC5791365; doi:10.1186/s12920-018-0319-0)
Supplement: Supplementary file 3 — Questionnaire administered at time point T3. (PDF 725 kb) [file 12920_2018_319_MOESM3_ESM.pdf]

PAPG Fall "Before" Questionnaire

The purpose of this study, entitled "Students' attitudes towards the use of personal genome data in the classroom", is to learn about students' attitudes towards having the option of analyzing their own genomes as part of the class process when learning about whole genome sequencing. Our goal in this research study is to learn more about how students feel about analyzing their own genome data in the classroom.

Your participation in this research study is voluntary. You may choose not to participate. If you choose to participate, you may stop taking part in this research study at any time without any penalty. This will not affect your participation, grade or any other aspect of your involvement in the personal genome analysis courses, or any other aspect of your education at Mount Sinai School of Medicine.

The procedure involves filling out an online survey that will take approximately 30 minutes. Your survey data will be identified only by a study number; your name and other information that could identify you will not be on the questionnaires. The study number will be "linked" to your name in a secure database which will not be accessible by any of the course instructors. This is to ensure that the instructors will not know if you are participating in the study, or what your answers to the questionnaires are.

If you have any questions, concerns, or complaints at any time about this research, or you think the research has hurt you, please contact Dr. Sanderson at telephone number 212-659-8520. This research has been reviewed and approved by Mount Sinai's Institutional Review Board. You may reach a representative of the Program for Protection of Human Subjects at Mount Sinai School of Medicine at telephone number (212) 824-8200 during standard work hours

1. Please select your choice below:

- ◯ I wish to continue with the questionnaire
- ◯ I DO NOT wish to continue and want to exit

PAPG Fall "Before" Questionnaire

Decision and decisional conflict

We are interested in knowing what your feelings are about analyzing your own versus an anonymous donated genome as part of this advanced whole genome sequencing course. We are interested in knowing what your feelings are about this at the present time.

2. Did you choose to get your genome sequenced as part of this course?

- ◯ No
- ◯ Yes
- ◯ Choose not to answer

Intention

3. Do you intend to use your personal genome for all of the analyses or just some of the analyses?

- All
- Some
- Unsure

If some, please specify which analyses you will use your own genome for.

4. Do you intend to exclude any regions from the analysis?

- No
- Yes
- Unsure

If yes, please tell us what types of information you plan to exclude.

PAPG Fall "Before" Questionnaire

5. Did you have a genetic counseling appointment prior to deciding whether to get your genome sequenced?

- ◯ No
- ◯ Yes
- ◯ Choose not to answer

6. Did you discuss whether or not to get your genome sequenced as part of this course with anyone?

- ◯ Yes
- ◯ No

7. If yes, who have you spoken to about whether or not to get your genome sequenced?

- ☐ Genetic counselor
- ☐ Physician or other health professional
- ☐ Mother
- ☐ Father
- ☐ Sibling(s)
- ☐ Other family member
- ☐ Friend(s)
- ☐ Spouse/significant other
- ☐ Course instructor(s)
- ☐ Other

If other, please specify:

8. As part of this study, you have been considering whether or not to receive your personal genome sequence data. The next questions are about your decision. Please indicate to what extent each statement is true for you AT THIS TIME by checking the appropriate boxes.

	Strongly disagree	Disagree	Neither agree nor disagree	Agree	Strongly agree
I am satisfied that I am adequately informed about the issues important to my decision.	◯	◯	◯	◯	◯
The decision I made was the best decision possible for me personally.	◯	◯	◯	◯	◯
I am satisfied that my decision was consistent with my personal values.	◯	◯	◯	◯	◯
I successfully carried out the decision I made.	◯	◯	◯	◯	◯
I am satisfied that this was my decision to make.	◯	◯	◯	◯	◯
I am satisfied with my decision.	◯	◯	◯	◯	◯

Reasons for and against using own genome

9. I think analyzing my own genome as part of an advanced whole genome sequencing course would be useful.

- ◯ Strongly disagree
- ◯ Disagree
- ◯ Neither agree nor disagree
- ◯ Agree
- ◯ Strongly agree

10. Reasons for using own genome:

	Strongly disagree	Disagree	Neither agree nor disagree	Agree	Strongly agree	Not applicable
Satisfy general curiosity	◯	◯	◯	◯	◯	◯
See if a specific disease runs in the family or is in DNA	◯	◯	◯	◯	◯	◯
Learn about genetic makeup without going through a physician	◯	◯	◯	◯	◯	◯
Inform family members about health risks	◯	◯	◯	◯	◯	◯
Understand what a patient may learn/experience	◯	◯	◯	◯	◯	◯
Help understand principles of human genetics	◯	◯	◯	◯	◯	◯

11. Reasons against using own genome:

	Strongly disagree	Disagree	Neither agree nor disagree	Agree	Strongly agree	Not applicable
Results are not reliable	◯	◯	◯	◯	◯	◯
Results are not accurate	◯	◯	◯	◯	◯	◯
Results are not predictive	◯	◯	◯	◯	◯	◯
Concern about privacy/risks to privacy	◯	◯	◯	◯	◯	◯
Information will not be medically useful/will not change medical decisions	◯	◯	◯	◯	◯	◯
Information will not help learn human genetics	◯	◯	◯	◯	◯	◯
Unwanted information	◯	◯	◯	◯	◯	◯
Costs too much	◯	◯	◯	◯	◯	◯

General views about whole genome sequencing

12. How useful do you think the results from whole genome sequencing will be to a physician?

- ◯ Not useful at all
- ◯ Not very useful
- ◯ Not sure
- ◯ Useful
- ◯ Very useful
- ◯ Not applicable

13. How useful do you think the results from whole genome sequencing information will be to patients themselves?

- ◯ Not useful at all
- ◯ Not very useful
- ◯ Not sure
- ◯ Useful
- ◯ Very useful
- ◯ Not applicable

14. How likely is it that knowing the results from whole genome sequencing for yourself would lead to any changes in your behavior?

- ◯ Not at all likely
- ◯ Not very likely
- ◯ Not sure
- ◯ Quite likely
- ◯ Very likely
- ◯ Not applicable

15. Please respond to the following statements

	Strongly disagree	Disagree	Neither agree nor disagree	Agree	Strongly agree	Not applicable
Whole genome sequencing is useful for patients.	○	○	○	○	○	○
If I underwent whole genome sequencing, I would ask a physician for help in interpreting the results.	○	○	○	○	○	○
Results of whole genome sequencing would influence my future health care decisions.	○	○	○	○	○	○
Physicians have a professional responsibility to help individuals understand the results they receive from whole genome sequencing, even if the physician has not ordered the test.	○	○	○	○	○	○
Physicians have enough knowledge to help individuals interpret results of whole genome sequencing.	○	○	○	○	○	○
Most people can accurately interpret whole genome sequencing results.	○	○	○	○	○	○
I know enough about genetics to understand the whole genome sequencing results.	○	○	○	○	○	○
I understand the risks and benefits of getting personal whole genome sequencing done.	○	○	○	○	○	○

PAPG Fall "Before" Questionnaire

Subjective understanding & self-efficacy

16. How would you describe your current understanding of genetics?

- ◯ None
- ◯ Minimal
- ◯ Some
- ◯ Moderate
- ◯ High

17. How would you rate your knowledge of genetics compared with others?

- ◯ Much less than others
- ◯ Less than others
- ◯ As much as others
- ◯ More than others
- ◯ Much more than others

18. How would you describe your current understanding of whole genome sequencing?

- ◯ None
- ◯ Minimal
- ◯ Some
- ◯ Moderate
- ◯ High

19. How would you rate your knowledge of whole genome sequencing compared with others?

- ◯ Much less than others
- ◯ Less than others
- ◯ As much as others
- ◯ More than others
- ◯ Much more than others

20. On a scale of 1-5, how confident are you in your ability to analyze and interpret whole genome sequence data?

- ◯ 1 No confidence
- ◯ 2
- ◯ 3 Moderate confidence
- ◯ 4
- ◯ 5 High confidence

Anxiety and depression

The questions on this page are designed to help us understand how you are feeling at the present time.

21. Please read the following statements which people have used to describe themselves. Please consider how you feel right now, that is, at this moment and respond with not at all, somewhat, moderately so, or very much so. There are no right or wrong answers. Do not spend too much time on any one statement but give the answer which seems to describe your present feelings best.

	Not at all	Somewhat	Moderately so	Very much so
I feel calm	◯	◯	◯	◯
I am tense	◯	◯	◯	◯
I feel upset	◯	◯	◯	◯
I am relaxed	◯	◯	◯	◯
I feel content	◯	◯	◯	◯
I am worried	◯	◯	◯	◯

22. Below is a list of a number of the ways you might have felt or behaved. Please check “Yes” or “No” if you have felt this way much of the time during the past week.

	Yes	No
I felt depressed.	◯	◯
I felt that everything I did was an effort.	◯	◯
My sleep was restless.	◯	◯
I was happy.	◯	◯
I felt lonely.	◯	◯
People were unfriendly.	◯	◯
I enjoyed life.	◯	◯
I felt sad.	◯	◯
I felt that people disliked me.	◯	◯
I could not get “going.”	◯	◯

Knowledge about personal genomic information

In this final section of the questionnaire, please read the following questions about genomics, and answer them as best you can. If you are not sure of an answer, don't worry, just check "I don't know".

23. You have a 37-year-old patient who has a family history of breast and ovarian cancer (her mother with bilateral breast cancer at the age of 45 years, her maternal aunt with ovarian cancer at the age of 52 years, and her maternal grandmother with bilateral breast cancer at the age of 50 years). Because she did not want her insurance company to discriminate against her, she participated in a research study offering results from whole genome sequencing. She wants you to help her understand her testing results so that she can undergo any appropriate screening and/or prophylactic surgeries.

As epidemiologic background, 13% of the population develops breast cancer in their lifetime, and 5–10% of cases of breast cancer are estimated to be due to a genetic predisposition.

The study promised to report all discovered pathogenic mutations in the 56 ACMG Incidental Findings genes, which includes BRCA1 and BRCA2 (two of several genes associated with hereditary breast and ovarian cancer). The study did not report any pathogenic mutations to your patient.

How would you best interpret this case? Check all that apply:

- ☐ Patient is affected with breast cancer
- ☐ Patient has average risk
- ☐ Patient has higher risk than average
- ☐ Patient has lower risk than average
- ☐ Patient is a carrier of breast cancer and may develop it
- ☐ Patient has no risk for breast cancer
- ☐ A different genetic test should be ordered
- ☐ I don't know how to interpret this case

24. Fundamental limitations in 2nd generation (e.g. Illumina HiSeq 2000) whole exome sequencing technology are? Check all that apply:

- [ ] Low read depth
- [ ] The high background rate of neutral mutation
- [ ] De novo mutations
- [ ] Important genomic regions aren't targeted
- [ ] Important variant types can't be detected
- [ ] I don't know the limitations of whole exome sequencing technology

25. You discover the same novel (i.e. not previously observed in large studies like 1000 Genomes) autosomal coding deletion in a repetitive portion of the genome in multiple (of 100) unrelated individuals participating in a whole exome sequencing study of a complex adult-onset neurodegenerative phenotype. The most likely conclusions are? Check all that apply:

- [ ] The individuals are actually related
- [ ] The variant is an artifact of the sequencing and analysis workflow
- [ ] The variant is causal for the phenotype of interest
- [ ] I don't know how to make any conclusions

26. During the analysis of the data from a whole exome sequencing test ordered for an affected child and their unaffected parents (e.g. a trio), you identify a novel de-novo missense mutation predicted to be benign by SIFT and Polyphen2, two functional prediction algorithms. Check all that apply:

- [ ] You expect to observe a variant like this by chance
- [ ] You don't expect to observe a variant like this by chance
- [ ] This variant could not be the cause of this child’s disease
- [ ] This variant could be the cause of this child’s disease
- [ ] I don't know how to interpret this variant

27. You discover a rare (0.1% global minor allele frequency) homozygous protein-coding variant that has been previously reported to be pathogenic for an adult-onset autosomal dominant condition in a child undergoing whole exome sequencing for an unrelated condition. The parents are unaffected and not related but of the same ethnic background. What is the best way to interpret this result? Check all that apply:

- [ ] The patient is at higher risk than other carriers of this mutation
- [ ] The child may descend from a bottlenecked population in which this variant is a founder mutation
- [ ] The two conditions are actually related in some way
- [ ] The original reports may be confounded by cryptic population stratification
- [ ] I don't know how to interpret these results

28. Your patient has a grandparent with macular degeneration. He is concerned about the chance he may develop it. About 3% of the population develops macular degeneration, and you learn that about 66% of the risk for macular degeneration is due to a genetic predisposition. The studies from which these variants were derived had 300-3,000 cases and 1,000-5,000 controls. The reported odds ratios were 1.14-3.4 and risk allele frequencies in controls between 12-95% depending on the SNP and study.

You review their genetic testing results and find the following: LOC387715-S69A, +-; CFH-intron, ++; CFB, ++; C2-E318D, ++; CFH-Y402H, +-; and C3-R80G, ++.

Presume that - represents the low-risk allele and + represents the at-risk allele.

How would you best interpret this case? Check all that apply:

- [ ] Patient is affected with macular degeneration
- [ ] Patient has average risk
- [ ] Patient has higher risk than average
- [ ] Patient has lower risk than average
- [ ] Patient is a carrier of macular degeneration and may develop it
- [ ] Patient has no risk for macular degeneration
- [ ] A different genetic test should be ordered
- [ ] I don't know how to interpret this case

29. Assume that sequencing reads are equally likely to be drawn from the paternal and maternal chromosome, and further assume that a minimum of 3 reads are needed from each chromosome to accurately call a heterozygous genotype. How would you calculate the probability of having enough reads to correctly call a heterozygous genotype that has 10-fold coverage?

- ◯ One (1) minus the binomial cumulative distribution function with n=10, p=0.5, and k=3
- ◯ The Poisson probability with k=3, lambda=10
- ◯ The sum of the binomial probability for k from 3 to 7 with n=10, p=0.5
- ◯ I don't know how to calculate this probability

30. You ask a colleague to run the whole genome data for a proband with an undiagnosed genetic disease through her ENSEMBL-based annotation pipeline and she reports a mutation that disrupts a splice-site acceptor that you did not detect in your RefSeq-based pipeline. What is the best the way to interpret these results? Check all that apply:

- [ ] Your colleague may have found the causal mutation
- [ ] RefSeq and ENSEMBL gene annotations are effectively the same so there is likely a bug in her pipeline
- [ ] The mutation your colleague found can’t be in a clinically relevant gene of known function
- [ ] The mutation likely lies in a transcript present in ENSEMBL that is not present in RefSeq
- [ ] I don't know how to interpret these results

31. The pipeline reports the following two heterozygous protein-coding variants in MLH1 in the whole genome sequence of a healthy research subject. Both protein-coding mutations are reported to be pathogenic for hereditary colorectal cancer. How could you best interpret this situation given the supplied information? Check all that apply (codon translation not required):

p.Lys618Glu (c.1852A>G, chr3:g.37089130A>G)

p.Lys618Thr (c.1853A>C, chr3:g.37089131A>C)

- [ ] A. This individual could be compound heterozygous, i.e. the protein-coding mutations are on different chromosomes
- [ ] B. This individual could carry both the Glu and Thr mutations in cis, i.e. both occur on the same chromosome
- [ ] C. This individual could be heterozygous for p.Lys618Ala (c.1852_1853delinsGC, chr3.g:37089130AA>GC)
- [ ] D. More than one of answers A-C (above) could be possible, and you will be unable to refine the interpretation using the NGS data
- [ ] E. More than one of answers A-C (above) could be possible, but all will ultimately have the same clinical interpretation
- [ ] F. I don't know how to interpret this data

32. Your 50-year old patient brings you a GWAS case-control study showing that their genotype is associated with a complex disease with an odds-ratio (OR) of 2.5. The disease has a prevalence of 25% and can arise from age 10 onwards. They are concerned that they have a 62.5% chance of developing the disease in the future. Which of the following is an accurate way to communicate your patient's risk to them given the available information? Check all that apply:

- [ ] You are actually underestimating your risk! Relative risk is usually larger than the odds-ratio.
- [ ] You are correct; you have a 62.5% chance of developing the disease in the future.
- [ ] You are correct; you are at 2.5-fold higher risk for the disease than the general population.
- [ ] You are overestimating your relative risk; your absolute risk to develop the disease will be above 25% but below 62.5%.
- [ ] You are overestimating both your relative risk and "pre-test" risk; we would estimate your absolute risk to develop the disease to be below 62.5% and may be below 25%.
- [ ] I don't know how to communicate their risk.

PAPG Fall "Before" Questionnaire

Comments

33. Finally, we are very interested in any additional thoughts or comments you might have regarding the possibility of analyzing personal genomes in an advanced whole genome sequencing course. Please write any suggestions, comments, concerns, thoughts or questions in the box below.

Thank you very much for taking the time to complete this questionnaire!
