# Supplementary material for: Impacts of incorporating personal genome sequencing into graduate genomics education: a longitudinal study over three course years
Source: BMC Med Genomics. 2018 Jan 30;11:5. doi: 10.1186/s12920-018-0319-0 (PMC5791365; doi:10.1186/s12920-018-0319-0)
Supplement: Supplementary file 4 — Questionnaire administered at time point T4. (PDF 791 kb) [file 12920_2018_319_MOESM4_ESM.pdf]

PAPG Fall "After" Questionnaire

The purpose of this study, entitled "Students' attitudes towards the use of personal genome data in the classroom", is to learn about students' attitudes towards having the option of analyzing their own genomes as part of the class process when learning about whole genome sequencing. Our goal in this research study is to learn more about how students feel about analyzing their own genome data in the classroom.

Your participation in this research study is voluntary. You may choose not to participate. If you choose to participate, you may stop taking part in this research study at any time without any penalty. This will not affect your participation, grade or any other aspect of your involvement in the personal genome analysis courses, or any other aspect of your education at the Icahn School of Medicine at Mount Sinai.

The procedure involves filling out an online survey that will take approximately 30 minutes. Your survey data will be identified only by a study number; your name and other information that could identify you will not be on the questionnaires. The study number will be "linked" to your name in a secure database which will not be accessible by any of the course instructors. This is to ensure that the instructors will not know if you are participating in the study, or what your answers to the questionnaires are.

If you have any questions, concerns, or complaints at any time about this research, or you think the research has hurt you, please contact Dr. Sanderson at saskia.sanderson@mssm.edu. This research has been reviewed and approved by Mount Sinai's Institutional Review Board. You may reach a representative of the Program for Protection of Human Subjects at Mount Sinai at telephone number (212) 824-8200 during standard work hours

1. Please select your choice below:

- ○ I wish to continue with the questionnaire
- ○ I DO NOT wish to continue and want to exit

PAPG Fall "After" Questionnaire

Decision, Decision Satisfaction, and Regret

We are interested in your feelings and experiences having analyzed your own or an anonymous donated genome as part of the "Practical Analysis of Your Personal Genome" course you took. We are interested in knowing what your feelings are <u>at the present time</u>.

2. Did you have the blood draw for whole genome sequencing as part of this course?

- ○ Yes
- ○ No
- ○ Choose not to answer

3. Did you analyze your own genome as part of this whole genome sequencing course?

- ○ Yes
- ○ No
- ○ Choose not to answer

4. Please reflect on the decision that you made about receiving or not receiving your own personal whole genome sequencing data as part of the course. Please show how strongly you agree or disagree with these statements by checking the box that best fits your views about your decision.

	Strongly disagree	Disagree	Neither agree or disagree	Agree	Strongly agree
It was the right decision	○	○	○	○	○
I regret the choice that was made	○	○	○	○	○
I would go for the same choice if I had to do it over again	○	○	○	○	○
The choice did me a lot of harm	○	○	○	○	○
The decision was a wise one	○	○	○	○	○

5. As part of this course, you considered whether or not to receive your personal genome sequencing data. The next questions are about your decision. Please indicate to what extent each statement is true for you <u>at the present time</u>.

	Strongly disagree	Disagree	Neither agree or disagree	Agree	Strongly agree
I am satisfied that I was adequately informed about the issues important to my decision.	○	○	○	○	○
The decision I made was the best decision possible for me personally.	○	○	○	○	○
I am satisfied that my decision was consistent with my personal values.	○	○	○	○	○
I successfully carried out the decision I made.	○	○	○	○	○
I am satisfied that this was my decision to make.	○	○	○	○	○
I am satisfied with my decision.	○	○	○	○	○

PAPG Fall "After" Questionnaire

Utility

6. I think analyzing my own genome as part of this whole genome sequencing course was useful

- Strongly agree
- Agree
- Neither agree or disagree
- Disagree
- Strongly disagree

7. Please respond to the following statements about how analyzing your own genome in class was or was not useful to you

	Strongly disagree	Disagree	Neither agree nor disagree	Agree	Strongly agree
I was more persistent in completing assignments or analyses because I used my own genome					
I better understand the patient experience because I used my own genome					
I learned useful health or personal information because I used my own genome					
I better understand genetics concepts because I used my own genome					
I performed more analyses outside of class because I used my own genome					
I was more thorough in my analyses because I used my own genome					

PAPG Fall "After" Questionnaire

Actual analyses

8. Did you use your personal genome sequencing data for all of the analyses discussed in class or just some of the analyses?

- All
- Some

If some, please specify which analyses you chose the reference genome for

9. Did you exclude any regions from analysis?

- No
- Yes

If yes, please tell us what type of information did you exclude

PAPG Fall "After" Questionnaire

Discussion of results

10. Did you receive any results from your whole genome sequencing that you felt were important to you?

- ◯ Yes
- ◯ No
- ◯ Not sure

11. If yes, which category or categories did the results fall into? Check as many as apply:

- ☐ Carrier status
- ☐ Pharmacogenomics
- ☐ Monogenic disease risk for me
- ☐ Physical appearance trait
- ☐ Complex polygenic disease risk
- ☐ Ancestry
- ☐ Variant(s) of unknown significance
- ☐ Other

If 'other', or if you would like to provide any more detail about your results please do so here

12. Have you discussed the results from your whole genome sequencing data with anyone?

- ◯ Yes
- ◯ No
- ◯ Choose not to answer

13. If yes, who have you talked to about your results from whole genome sequencing data? Check all that apply:

- [ ] Genetic counselor
- [ ] Physician or other health professional
- [ ] Mother
- [ ] Father
- [ ] Sibling
- [ ] Other family member
- [ ] Friend(s)
- [ ] Spouse/significant other
- [ ] Course instructor(s)
- [ ] Other (please specify)

14. If you did not discuss the results from your genome sequencing data with your family members, why not? Check all that apply:

- [ ] I don't feel that my results are important enough to share.
- [ ] I don't think family members are interested in my results.
- [ ] I am concerned about how my family members would react to my results.
- [ ] I plan to discuss my results with family members but haven't gotten around to it.
- [ ] Other (please specify)

15. If you did <u>not</u> discuss the results from your genome sequencing data with a health professional, why not? Check all that apply:

- [ ] I would have concerns about approaching a healthcare professional with the results of my analyses.
- [ ] I would have concerns about my genomic information being incorporated into my medical record.
- [ ] I do not feel that my results are important enough to share.
- [ ] I plan to discuss my results with a healthcare practitioner but have not gotten around to it.
- [ ] I am confident in my ability to understand my genome sequencing results without the aid of a healthcare professional.
- [ ] I do not think my healthcare professional is knowledgeable enough about genomic sequencing to incorporate my results into my medical care.
- [ ] I did not receive any results that require medical follow-up.
- [ ] Other (please specify)

MICRA

16. The questions below are about some specific responses you may have had after analyzing your personal whole genome sequence data. Please answer every question regardless of what results you obtained through your analysis of your sequence data. Please indicate whether you have experienced each statement never, rarely, sometimes, or often in the past week.

	Never	Rarely	Sometimes	Often
Feeling upset about my whole genome sequencing results	○	○	○	○
Feeling sad about my whole genome sequencing results	○	○	○	○
Feeling anxious or nervous about my whole genome sequencing results	○	○	○	○
Feeling guilty about my whole genome sequencing results	○	○	○	○
Feeling relieved about my whole genome sequencing results	○	○	○	○
Feeling happy about my whole genome sequencing results	○	○	○	○
Feeling a loss of control	○	○	○	○
Having problems enjoying life because of my whole genome sequencing results	○	○	○	○
Worrying about my risk of getting a specific disease	○	○	○	○
Being uncertain about what my whole genome sequencing results mean about my disease risk	○	○	○	○
Being uncertain about what my whole genome sequencing results mean for my child(ren) and/or family's disease risk	○	○	○	○
Having difficulty making decisions about disease screening or prevention (e.g., having preventive surgery or getting medical tests done)	○	○	○	○
Understanding clearly my choices for disease prevention or early detection	○	○	○	○
Feeling frustrated that there are no definite disease prevention guidelines for me	○	○	○	○
Thinking about my whole genome sequencing results has affected my work or family life	○	○	○	○
Feeling concerned about how my whole genome sequencing results will affect my insurance status	○	○	○	○

	Never	Rarely	Sometimes	Often
Having difficulty talking about my whole genome sequencing results with family members	○	○	○	○
Feeling that my family has been supportive during the whole genome sequencing process	○	○	○	○
Feeling satisfied with family communication about my whole genome sequencing results	○	○	○	○
Worrying that the whole genome sequencing process has brought about conflict within my family	○	○	○	○
Feeling regret about getting my whole genome sequencing results	○	○	○	○

17. If you have children please indicate whether you have experienced each statement never, rarely, sometimes, or often **in the past week**.

	Never	Rarely	Sometimes	Often
Worrying about the possibility of my children getting a specific disease	○	○	○	○
Feeling guilty about possibly passing on the disease risk to my child(ren)	○	○	○	○

PAPG Fall "After" Questionnaire

General attitudes about WGS

18. How useful do you think the results from whole genome sequencing will be to a physician?

- ◯ Not useful at all
- ◯ Not very useful
- ◯ Not sure
- ◯ Useful
- ◯ Very useful

19. How useful do you think the results from whole genome sequencing information will be to patients themselves?

- ◯ Not useful at all
- ◯ Not very useful
- ◯ Not sure
- ◯ Useful
- ◯ Very useful

20. How likely is it that knowing the results from whole genome sequencing for yourself would lead to any changes in your behavior?

- ◯ Not at all likely
- ◯ Not very likely
- ◯ Not sure
- ◯ Quite likely
- ◯ Very likely

21. Please respond to the following statements

	Strongly Disagree	Disagree	Neither agree nor disagree	Agree	Strongly agree	N/A
Whole genome sequencing is useful for patients.	○	○	○	○	○	○
Physicians have a professional responsibility to help individuals understand the results they receive from whole genome sequencing, even if the physician has not ordered the test.	○	○	○	○	○	○
Physicians have enough knowledge to help individuals interpret results of whole genome sequencing.	○	○	○	○	○	○
Most people can accurately interpret whole genome sequencing results	○	○	○	○	○	○
I know enough about genetics to understand the whole genome sequencing results	○	○	○	○	○	○
I understand the risks and benefits of using/getting personal whole genome sequencing done	○	○	○	○	○	○

Reasons for and against using own genome

Having been offered personal genome sequencing as an optional part of an advanced whole genome sequencing class, please respond to the following statements:

22. Reasons for using own genome:

	Strongly disagree	Disagree	Neither agree nor disagree	Agree	Strongly agree	Not applicable
Satisfy general curiosity	○	○	○	○	○	○
See if a specific disease runs in the family or is in DNA	○	○	○	○	○	○
Learn about genetic makeup without going through a physician	○	○	○	○	○	○
Inform family members about health risks	○	○	○	○	○	○
Understand what a patient may learn/experience	○	○	○	○	○	○
Help understand principles of human genetics	○	○	○	○	○	○

23. Reasons against using own genome:

	Strongly disagree	Disagree	Neither agree nor disagree	Agree	Strongly agree	Not applicable
Results are not reliable	○	○	○	○	○	○
Results are not accurate	○	○	○	○	○	○
Results are not predictive	○	○	○	○	○	○
Concern about privacy/risks to privacy	○	○	○	○	○	○
Information will not be medically useful/will not change medical decisions	○	○	○	○	○	○
Information will not help learn human genetics	○	○	○	○	○	○
Unwanted information	○	○	○	○	○	○
Costs too much	○	○	○	○	○	○

PAPG Fall "After" Questionnaire

Subjective understanding & self-efficacy

24. How would you describe your current understanding of genetics?

- None
- Minimal
- Some
- Moderate
- High

25. How would you rate your knowledge of genetics compared with others?

- Much less than others
- Less than others
- As much as others
- More than others
- Much more than others

26. How would you describe your current understanding of whole genome sequencing?

- None
- Minimal
- Some
- Moderate
- High

27. How would you rate your knowledge of whole genome sequencing compared with others?

- Much less than others
- Less than others
- As much as others
- More than others
- Much more than others

28. On a scale of 1-5, how confident are you in your ability to analyze and interpret whole genome sequence data?

- ◯ 1 No confidence
- ◯ 2
- ◯ 3 Moderate confidence
- ◯ 4
- ◯ 5 High confidence

PAPG Fall "After" Questionnaire

Impact of course

29. Has taking this course had any impact on members of your family for any reason?

- ◯ Yes
- ◯ No
- ◯ Not sure

30. If yes, can you explain how?

31. Have you applied the knowledge that you gained from the course in any of your studies, clinical practice or research work?

- ◯ Yes
- ◯ No
- ◯ Not sure

32. If yes, in what ways? Check all that apply:

- ☐ Use of online databases or tools such as UCSC Genome Browser and HGMD
- ☐ Computing skills such as using the Minerva compute cluster
- ☐ Variant interpretation
- ☐ Communicating the capabilities and limitations of next-generation sequencing technology
- ☐ Application of the genome analysis pipeline
- ☐ Other (please specify)

Anxiety and depression

The questions on this page are designed to help us understand how you are feeling at the present time.

33. Please read the following statements which people have used to describe themselves. Please consider how you feel right now, that is, at this moment. There are no right or wrong answers. Do not spend too much time on any one statement but give the answer which seems to describe your present feelings best.

	Not at all	Somewhat	Moderately so	Very much so
I feel calm	○	○	○	○
I am tense	○	○	○	○
I feel upset	○	○	○	○
I am relaxed	○	○	○	○
I feel content	○	○	○	○
I am worried	○	○	○	○

34. Below is a list of a number of ways you might have felt or behaved. Please check "Yes" or "No" if you this way much of the time during the past week.

	Yes	No
I felt depressed	○	○
I felt that everything I did was an effort	○	○
My sleep was restless	○	○
I was happy	○	○
I felt lonely	○	○
People were unfriendly	○	○
I enjoyed life	○	○
I felt sad	○	○
I felt that people disliked me	○	○
I could not get "going"	○	○

PAPG Fall "After" Questionnaire

Engagement

Please answer the following questions regardless of whether you analyzed your own or a reference genome.

35. How many variants do you estimate you analyzed outside of the specific course assignments?

- ◯ 0
- ◯ 1-2
- ◯ 3-5
- ◯ 6-10
- ◯ 11-20
- ◯ 21-30
- ◯ More than 30

36. How many hours do you estimate you spent outside of class assignments analyzing your genome?

- ◯ Less than 1 hour
- ◯ 1-2 hours
- ◯ 2-5 hours
- ◯ 5-10 hours
- ◯ 10-20 hours
- ◯ 20-30 hours
- ◯ More than 30 hours

PAPG Fall "After" Questionnaire

Objective Understanding

37. You have a 37-year-old patient who has a family history of breast and ovarian cancer (her mother with bilateral breast cancer at the age of 45 years, her maternal aunt with ovarian cancer at the age of 52 years, and her maternal grandmother with bilateral breast cancer at the age of 50 years). Because she did not want her insurance company to discriminate against her, she participated in a research study offering results from whole genome sequencing. She wants you to help her understand her testing results so that she can undergo any appropriate screening and/or prophylactic surgeries.

As epidemiologic background, 13% of the population develops breast cancer in their lifetime, and 5-10% of cases of breast cancer are estimated to be due to a genetic predisposition.

The study promised to report all discovered pathogenic mutations in the 56 ACMG Incidental Findings genes, which includes BRCA1 and BRCA2 (two of several genes associated with hereditary breast and ovarian cancer). The study did not report any pathogenic mutations to your patient.

How would you best interpret this case? Check all that apply:

- [ ] Patient is affected with breast cancer
- [ ] Patient has average risk
- [ ] Patient has higher risk than average
- [ ] Patient has lower risk than average
- [ ] Patient is a carrier of breast cancer and may develop it
- [ ] Patient has no risk for breast cancer
- [ ] A different genetic test should be ordered
- [ ] I don't know how to interpret this case

38. Fundamental limitations in 2nd generation (e.g. Illumina HiSeq 2000) whole exome sequencing technology are? Check all that apply:

- [ ] Low read depth
- [ ] The high background rate of neutral mutation
- [ ] De novo mutations
- [ ] Important genomic regions aren't targeted
- [ ] Important variant types can't be detected
- [ ] I don't know the limitations of whole exome sequencing technology

39. You discover the same novel (i.e. not previously observed in large studies like 1000 Genomes) autosomal coding deletion in a repetitive portion of the genome in multiple (of 100) unrelated individuals participating in a whole exome sequencing study of a complex adult-onset neurodegenerative phenotype. The most likely conclusions are? Check all that apply:

- [ ] The individuals are actually related
- [ ] The variant is an artifact of the sequencing and analysis workflow
- [ ] The variant is causal for the phenotype of interest
- [ ] I don't know how to make any conclusions

40. During the analysis of the data from a whole exome sequencing test ordered for an affected child and their unaffected parents (e.g. a trio), you identify a novel de-novo missense mutation predicted to be benign by SIFT and Polyphen2, two functional prediction algorithms. Check all that apply:

- [ ] You expect to observe a variant like this by chance
- [ ] You don't expect to observe a variant like this by chance
- [ ] This variant could not be the cause of this child's disease
- [ ] This variant could be the cause of this child's disease
- [ ] I don't know how to interpret this variant

41. You discover a rare (0.1% global minor allele frequency) homozygous protein-coding variant that has been previously reported to be pathogenic for an adult-onset autosomal dominant condition in a child undergoing whole exome sequencing for an unrelated condition. The parents are unaffected and not related but of the same ethnic background. What is the best way to interpret this result? Check all that apply:

- [ ] The patient is at higher risk than other carriers of this mutation
- [ ] The child may descend from a bottlenecked population in which this variant is a founder mutation
- [ ] The two conditions are actually related in some way
- [ ] The original reports may be confounded by cryptic population stratification
- [ ] I don't know how to interpret these results

42. Your patient has a grandparent with macular degeneration. He is concerned about the chance he may develop it. About 3% of the population develops macular degeneration, and you learn that about 66% of the risk for macular degeneration is due to a genetic predisposition. The studies from which these variants were derived had 300-3,000 cases and 1,000-5,000 controls. The reported odds ratios were 1.14-3.4 and risk allele frequencies in controls between 12-95% depending on the SNP and study.

You review their genetic testing results and find the following: LOC387715-S69A, +-; CFH-intron, ++; CFB, ++; C2-E318D, ++; CFH-Y402H, +-; and C3-R80G, ++.

Presume that - represents the low-risk allele and + represents the at-risk allele.

How would you best interpret this case? Check all that apply:

- [ ] Patient is affected with macular degeneration
- [ ] Patient has average risk
- [ ] Patient has higher risk than average
- [ ] Patient has lower risk than average
- [ ] Patient is a carrier of macular degeneration and may develop it
- [ ] Patient has no risk for macular degeneration
- [ ] A different genetic test should be ordered
- [ ] I don't know how to interpret this case

43. Assume that sequencing reads are equally likely to be drawn from the paternal and maternal chromosome, and further assume that a minimum of 3 reads are needed from each chromosome to accurately call a heterozygous genotype. How would you calculate the probability of having enough reads to correctly call a heterozygous genotype that has 10-fold coverage?

- ○ One (1) minus the binomial cumulative distribution function with n=10, p=0.5, and k=3
- ○ The Poisson probability with k=3, lambda=10
- ○ The sum of the binomial probability for k from 3 to 7 with n=10, p=0.5
- ○ I don't know how to calculate this probability

44. You ask a colleague to run the whole genome data for a proband with undiagnosed genetic disease through her ENSEMBL-based annotation pipeline and she reports a mutation that disrupts a splice-site acceptor that you did not detect in your RefSeq-based pipeline. What is the best the way to interpret these results? Check all that apply:

- [ ] Your colleague may have found the causal mutation
- [ ] RefSeq and ENSEMBL gene annotations are effectively the same so there is likely a bug in her pipeline
- [ ] The mutation your colleague found can't be in a clinically relevant gene of known function
- [ ] The mutation likely lies in a transcript present in ENSEMBL that is not present in RefSeq
- [ ] I don't know how to interpret these results

45. The pipeline reports the following two heterozygous protein-coding variants in MLH1 in the whole genome sequence of a healthy research subject. Both protein-coding mutations are reported to be pathogenic for hereditary colorectal cancer. How could you best interpret this situation given the supplied information? Check all that apply (no codon translation should be required):

p.Lys618Glu (c.1852A>G, chr3:g.37089130A>G)
p.Lys618Thr (c.1853A>C, chr3:g.37089131A>C)

- [ ] A. This individual could be compound heterozygous, i.e. the protein-coding mutations are on different chromosomes
- [ ] B. This individual could carry both the Glu and Thr mutations in cis, i.e. both occur on the same chromosome
- [ ] C. This individual could be heterozygous for p.Lys618Ala (c.1852_1853delinsGC, chr3.g:37089130AA>GC)
- [ ] D. More than one of answers A-C could be possible, and you will be unable to refine the interpretation using the NGS data
- [ ] E. More than one of answers A-C could be possible, but all will ultimately have the same clinical interpretation
- [ ] F. I don't know how to interpret this data

46. Your 50-year old patient brings you a GWAS case-control study showing that their genotype is associated with a complex disease with an odds-ratio (OR) of 2.5. The disease has a prevalence of 25% and can arise from age 10 onwards. They are concerned that they have a 62.5% chance of developing the disease in the future. Which of the following is an accurate way to communicate your patient's risk to them given the available information? Check all that apply:

- [ ] You are actually underestimating your risk! Relative risk is usually larger than the odds-ratio.
- [ ] You are correct; you have a 62.5% chance of developing the disease in the future.
- [ ] You are correct; you are at 2.5-fold higher risk for the disease than the general population.
- [ ] You are overestimating your relative risk; your absolute risk to develop the disease will be above 25% but below 62.5%.
- [ ] You are overestimating both your relative risk (RR) and "pre-test" risk; we would estimate your absolute risk to develop the disease to be below 62.5% and may be below 25%.
- [ ] I don't know how to communicate their risk.

PAPG Fall "After" Questionnaire

Comments

47. Finally, we are very interested in any additional thoughts or comments you might have regarding the possibility of analyzing personal genomes in an advanced whole genome sequencing course. Please write any suggestions, comments, concerns, thoughts or questions in the box below.

Thank you very much for taking the time to complete this questionnaire!
